# Structural and energetic analyses of SARS-CoV-2 *N*-terminal domain characterise sugar binding pockets and suggest putative impacts of variants on COVID-19 transmission

**DOI:** 10.1016/j.csbj.2022.11.004

**Published:** 2022-11-07

**Authors:** Su Datt Lam, Vaishali P. Waman, Franca Fraternali, Christine Orengo, Jonathan Lees

**Affiliations:** a Institute of Structural and Molecular Biology, University College London, London, United Kingdom; b Department of Applied Physics, Faculty of Science and Technology, Universiti Kebangsaan Malaysia, Bangi, Malaysia; c Translational Health Sciences, Bristol Medical University, University of Bristol, Bristol, United Kingdom; d Faculty of Health and Life Sciences, Oxford Brookes University, Oxford, United Kingdom

**Keywords:** SARS-CoV-2, Sialic acid-binding pocket, Spike *N*-terminal domain, Structural and functional impacts, Variants of concern

## Abstract

Coronavirus disease 2019 (COVID-19) caused by SARS-CoV-2 is an ongoing pandemic that causes significant health/socioeconomic burden. Variants of concern (VOCs) have emerged affecting transmissibility, disease severity and re-infection risk. Studies suggest that the - *N*-terminal domain (NTD) of the spike protein may have a role in facilitating virus entry via sialic-acid receptor binding. Furthermore, most VOCs include novel NTD variants. Despite global sequence and structure similarity, most sialic-acid binding pockets in NTD vary across coronaviruses. Our work suggests ongoing evolutionary tuning of the sugar-binding pockets and recent analyses have shown that NTD insertions in VOCs tend to lie close to loops.

We extended the structural characterisation of these sugar-binding pockets and explored whether variants could enhance sialic acid-binding. We found that recent NTD insertions in VOCs (i.e., Gamma, Delta and Omicron variants) and emerging variants of interest (VOIs) (i.e., Iota, Lambda and Theta variants) frequently lie close to sugar-binding pockets. For some variants, including the recent Omicron VOC, we find increases in predicted sialic acid-binding energy, compared to the original SARS-CoV-2, which may contribute to increased transmission. These binding observations are supported by molecular dynamics simulations (MD).

We examined the similarity of NTD across Betacoronaviruses to determine whether the sugar-binding pockets are sufficiently similar to be exploited in drug design. Whilst most pockets are too structurally variable, we detected a previously unknown highly structurally conserved pocket which can be investigated in pursuit of a generic pan-Betacoronavirus drug. Our structure-based analyses help rationalise the effects of VOCs and provide hypotheses for experiments. Our findings suggest a strong need for experimental monitoring of changes in NTD of VOCs.

## Graphical abstract

**Figure:**
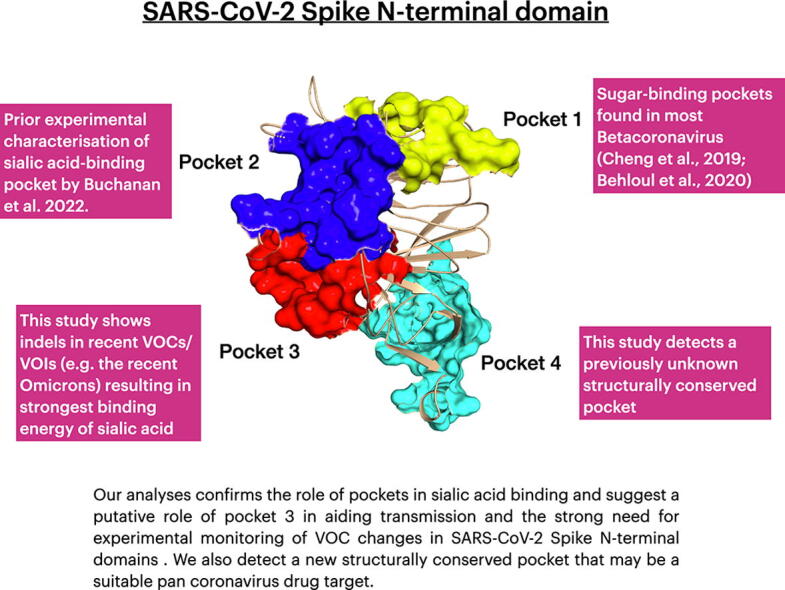

## 1 Introduction

Coronavirus disease 2019 (COVID-19), first detected in December 2019, is an ongoing pandemic situation and a cause of significant health and socioeconomic burden globally. COVID-19 is caused by a novel coronavirus namely SARS-CoV-2 [1], [2], [3], which has led to the loss of the lives of around 6.5 million people worldwide [4].

Vaccines are being successfully administered, however the emergence of new variants of concern (VOCs) raises key questions about their phenotypic impact on transmissibility, disease severity, risk of re-infection and impact on diagnostics [5], [6]. Currently, the mutation rate of SARS-CoV-2 is estimated to be between 0.5 × 10^-3^ − 1.1 × 10^-3^ substitutions/site/year [7], [8], [9]. So far, five VOCs have been detected namely: Alpha variant (reported in 203 countries), Beta (153 countries), Gamma (113 countries), Delta (in 208 countries) and Omicron variant (in 194 countries) [10]. All VOCs are reported to carry novel mutations particularly in the domains of their spike protein, along with a few additional mutations in other parts of the virus genome (https://sars2.cvr.gla.ac.uk/cog-uk/; https://www.who.int/en/activities/tracking-SARS-CoV-2-variants/).

The Spike protein (S) is the key antigen and plays a major role in virus-host cell attachment and entry and fusion with the host cell membrane. Spike protein is both a point mutation and a recombination hotspot [11], [12] and it is the primary target for neutralising antibodies during infection and the majority of vaccines. It is a homo-trimeric protein and in SARS-CoV-2, it is made up of two subunits, S1 and S2 [13]. The S1 subunit comprises two domains: the NTD (*N*-terminal domain) and the RBD (receptor binding domain). Structural studies have revealed mechanisms by which the RBD of the SARS-CoV-2 spike protein interacts with human ACE2 protein to gain entry into the host cell [14], [15].

A recent study by Dicken and co-workers (2021) showed that the *N*-terminal domain (NTD) of the spike protein might also have a role in facilitating efficient virus entry [16]. Their experimental study of the Alpha variant showed that amino acid deletions in residues 69 and 70 of NTD resulted in more efficient entry into human cells and infection, as compared to the reference strain (Wuhan-1). Furthermore, the NTD domain is observed to be a sequence diversity hotspot and a comprehensive sequence-based analyses on the Sarbecovirus subgenus of the Betacoronaviruses (BCoVs) showed five distinct regions of insertions and deletions (indels) in the NTD domains of different BCoVs [17]. Garry et al. (2021) suggested that these probably occur in loops [17]. Intriguingly, indel regions 1, 3 and 5 correspond to an NTD antigenic supersite [18] (see Fig. 4B), that is a target of all known NTD-specific neutralising antibodies, thus supporting the role of NTD in eliciting protective immunity. McCallum et al. (2020) recently revealed that most of the VOCs harbour novel mutations in this NTD antigenic supersite [18].

The *N*-terminal domain of SARS-CoV-2 Spike protein has a beta sandwich structure and belongs to the “Spike glycoprotein, *N*-terminal domain” superfamily (ID: 2.60.120.960) in the CATH structural classification [19], [20]. The sugar-binding protein human Galectin is also found within the same fold group (2.60.120) and the two domains are structurally similar. Studies have shown that the NTD of HKU-23 (another BCoV) is structurally related to the Galectin fold (e.g., Galectin-3), possibly co-opted by the virus from the host [21]. Furthermore, the role of NTD in binding to sialic acid-containing sugars like glycoproteins and gangliosides has been reported in several coronaviruses and other viruses [22], [23]. There is also some evidence from experimental studies from other BCoVs that changes in NTD may improve affinity for sugars on the host cell surface and therefore increase the affinity of the virus for the specific host cell [24], [25]. Furthermore, Amraei et al. (2021) proposed that the NTD domain of the Spike protein in SARS-CoV-2 could bind to C-type lectin receptors such as L-SIGN/CD-SIGN using various biochemical assays [26].

More recently, Cheng et al. (2019) analysed experimental 3D structures of BCoVs (Bovine CoV-NTD, PHEV-NTD, HCoV-OC43-NTD, HCoV-HKU23-NTD, HKU1-NTD, MHV-NTD, SARS-CoV-NTD and MERS-NTD) and showed via binding assays that they could bind to sugars such as sialic acid-containing glycoproteins and gangliosides in an equivalent binding pocket to Galectin-3 (referred to as ‘Pocket 1’) [27]. Interestingly, Behloul and co-workers (2020) found a GTNGTKR motif, a known sugar-binding motif (located at residues 72–78 in SARS-CoV-2 Spike protein) within a different pocket on the protein (referred to as ‘Pocket 2’) suggesting that this region may also bind sugars [28]. Similarly, Awasthi et al. (2020) performed a comparative structural analysis of Pocket 2 in the NTD of SARS-CoV-2 and SARS-CoV, using molecular dynamics and docking approaches [29]. Their analyses highlighted the flexible nature of the loop (L244-G261, part of Pocket 2) allowing Pocket 2 of SARS-CoV-2:NTD to bind sialosides more strongly than that of SARS-CoV [29]. Pocket 2 is also detected in other coronaviruses including BovineCoV-NTD, PHEV-NTD, HCoV-OC43-NTD, HCoV-HKU23-NTD, HKU1-NTD, MHV-NTD but not well defined in SARS-CoV-NTD nor MERS-NTD [27]. Cheng et al. [16] showed that this pocket also had the capacity to bind sugars, except for SARS-CoV. Furthermore, the structure of HCoV-OC43 has been determined with a sialic acid (i.e., 9-O-acetyl-sialic acid) bound in Pocket 2 [30]. Recently, a third druggable pocket was detected computationally in the NTD domain of SARS-CoV-2 (Pocket 3) [31]. Bò and co-workers (2021) performed MD simulation of this pocket and suggested this pocket may also bind sialic acid [32].

Recently, experimental studies have confirmed the predicted binding of sialic sugar to NTD in SARS-CoV-2. For example, a recent study used a glyconanoparticle platform to investigate the spike protein's ability to attach to neuraminic acid and found persistent binding, showing SARS-CoV-2 spike glycan-binding activity [33]. Similarly, Unione et al. (2022) performed Saturation Transfer Difference (STD NMR) experiment and also reported sialic acid-binding in NTD of SARS-CoV-2 [34]. More directly, Buchanan et al. (2022) performed a universal saturation transfer analysis and also a HADDOCK docking analysis and reported sialic acid-binding in Pocket 2 [35].

Our work further explores the role of NTD of SARS-CoV-2 in sialic acid binding. We used docking studies to examine the characteristics of all three pockets and also detected a fourth pocket (Pocket 4). Since Awasthi et al. (2020) showed that insertions in SARS-CoV-2 relative to SARS-CoV enhanced sialic binding, we structurally compared all 4 pockets across related coronaviruses to determine whether (and if so how) these pockets had evolved to enhance binding to polysaccharides [29]. Our results support the work of Awasthi et al. (2020), showing that SARS-CoV-2 possesses additional loops in Pocket 2 that extend the pocket to increase contact with polysaccharides [29]. We also found extensions in Pocket 3. We observe that Pockets 2 and 3 bind sialic acid with comparable binding energies, and more strongly than Pocket 1 or Pocket 4.

We subsequently determined whether variants, found in VOCs and VOIs of SARS-CoV-2, including insertions/deletions in the loops defining the NTD sugar binding pockets could result in enhanced binding of the spike protein to sialic acid (and therefore to the host membrane). We observe that in recent VOCs, like Omicron, variants increase the binding of sialic acids to Pocket 3 which may help to enhance infection by the virus and therefore transmission of the disease. These observations are supported by MD simulations.

Finally, we assessed which of the pockets were druggable and sufficiently structurally conserved across related coronaviruses for their structural features to be exploited in the design of generic drugs against a wide range of BCoV viruses. Whilst there are some therapeutic strategies targeted at Pocket 1 in SARS-CoV [22], this pocket is too variable across BCoVs. Pockets 2 and 3 are even more structurally variable and therefore not amenable to pan-BCoV drug design. However, the fourth druggable pocket, Pocket 4, appears to be highly structurally conserved and could therefore be further investigated in pursuit of a generic (pan-BCoVs) drug. Sialic acid targeting, or mimicking drugs could serve as good candidates for antiviral strategies [36].

## 2 Materials and methods

### 2.1 Sequence data

To explore the sequence and structure conservation of BCoVs, we selected a set of representative sequences, expected to be closely related to SARS-CoV-2 and also strains that have caused human disease [37]. We obtained 36 nucleotide sequences of the Sarbecovirus subgenus of the BCoV and SARS-related coronaviruses from NCBI [38] and GISAID [39], [40], [41] databases (See Supplementary Table 1). We extracted the Spike protein sequences by scanning the sequence of the SARS-CoV-2 Spike protein (YP_009724390.1) against the nucleotide database of BCoV sequences using NCBI BLAST v2.6 tblastn [42]. For each of the Spike protein sequences extracted, we then extracted the *N*-terminal domain (residues 1–303). We also obtained BCoV NTD domain sequences for these proteins from the CATH “Spike glycoprotein, *N*-terminal domain” domain superfamily (ID: 2.60.120.960) [19].

### 2.2 Structure data

We obtained experimental structures for the NTD-domain of SARS-CoV (PDB ID: 6ACC), Pangolin-CoV-GX (PDB ID: 7CN8), Pangolin-CoV-GD (PDB ID: 7BBH), Bat-CoV-RaTG13 (PDB ID: 7CN4), SARS-COV-2 (PDB ID: 7C2L and PDB ID: 6ZGE), HCoV-OC43 (PDB ID: 6NZK) and Human Galectin-3 (PDB ID: 1A3K) from the Protein Data Bank (PDB) [43].

Structural models of other BCoV NTDs were built using an in-house FunMOD modelling pipeline [44], [45] which exploits the MODELLER method [46], provided they had >40% sequence identity to the template (see Supplementary Table 2 for details). Otherwise, models were built using AlphaFold2 ([47], ‘AlphaFold2_mmseqs2′ notebook available from https://github.com/sokrypton/ColabFold, [48]) (see Supplementary Table 3 for details). Using HH-suite version 3 [49], FunMOD produced query–template alignments that were then fed into the MODELLER v.9.23 program [46]. The ‘very_slow’ schedule was employed for model refinement. For each query, ten models were generated and we then chose the model with the lowest normalised DOPE score (nDOPE) [50], which measures the quality of the model. Positive scores are likely to be poor models, while scores lower than -1 are likely to be native-like.

Since performing our analyses the AlphaFold2 structural modelling method was published and found to give higher quality than models from other programs for proteins with no close homologues in the PDB [47], [51]. However, for proteins with close homologues (> 40% sequence identity), the models are comparable. All the domains we modelled with FunMOD had > 45% sequence identity. Nevertheless, we tested whether using AlphaFold2 models would change our results. For the lowest sequence identity (BatCoV-RmYN02, query-template sequence identity of 47%), we tested the value by using a model generated by AlphaFold2 instead. We found that the models built by the two methods did not differ significantly (SSAP score of 89.8 (out of 100) and RMSD value of 1.73). We also docked sialic acid into both models and found the PRODIGY predicted binding energy to be highly similar (differ by only 0.1 kcal/mol).

### 2.3 Multiple sequence alignment and phylogenetic tree

To build a Maximum Likelihood tree, we aligned all NTD amino acid sequences using CLUSTAL-OMEGA [52]. A phylogenetic tree was inferred according to the Maximum Likelihood method. Genetic distance was computed using the Whelan and Goldman model [53] and gamma-distributed rate variation among sites (WAG + G). The evolutionary history was inferred using the Maximum Likelihood method and the analyses were conducted using MEGA11 [54].

We used ESPript3 web server (http://espript.ibcp.fr/ESPript/ESPript/) [55] to produce structure-based multiple sequence alignment. The secondary structure elements were defined based on the SARS-CoV-2 NTD structure (PDB ID 6ZGE).

The ScoreCons method [56] was used to calculate the sequence conservation of residues at a particular position. ScoreCons reports a score between 0 and 1 which is robust for multiple sequence alignments with high information content (DOPS score >70). A ScoreCons value above 0.7 suggests a highly conserved position.

### 2.4 Structure comparison and structural analysis

Protein structures were compared using our in-house SSAP algorithm [57]. The SSAP score ranges from 0 to 100. Structures with a SSAP score above 80 are considered to be highly similar. Protein structures were rendered using PyMOL [58] and UCSF Chimera [59].

We used mCSM-PPI2 [60] to investigate the effect of mutations on the stability of the complex between SARS-CoV-2 Spike RBD domain and human ACE2 (using PDB ID 7A95).

### 2.5 Druggable pocket prediction and molecular docking

We used CavityPlus [61] to predict druggable pockets using chain A of SARS-CoV-2 structure (PDB ID: 7C2L). The CavityPlus DrugScore denotes the druggability of a particular pocket with more positive scores indicating more druggable sites.

Molecular docking was done using the HADDOCK 2.4 webserver (https://wenmr.science.uu.nl/haddock2.4/) [62]. HADDOCK is one of the top-performing protein–ligand binding methods in the D3R Grand Challenge 2 and 3 [63], [64]. The sialic acid used was 9-O-acetylated sialic acid (PubChem ID 71312953) [65]. This ligand is the sialic acid bound to the experimental structure of HCoV-OC43 (PDB ID 6NZK [30]). The HADDOCK score is a linear combination of van der Waals, electrostatics, and desolvation energy terms. A lower HADDOCK score signifies stronger binding. The binding affinity of sialic acid-NTD was predicted using HADDOCK’s PRODIGY-LIGAND server (https://wenmr.science.uu.nl/prodigy/) [66]. We calculated the binding constant K_b_ using ΔG = -RT ln K_b_, where R is the gas constant (1.987 cal·K^-^^1^·mol^-1^), T is temperature (298 K). We used the LIGPLOT+ program of PDBsum [67] to examine residue interactions between sialic acid and the NTDs.

To perform mutagenesis analyses on the residues interacting with sialic acid we generated a mutant by mutating the respective residue to amino acid alanine. We then docked the sialic acid to the same region using HADDOCK and calculated the PRODIGY binding energy.

### 2.6 Molecular dynamics simulation

We used GROMACS 2020.6 [68] to perform simulations on the studied systems. Protein topologies were created using the CHARMM-36 force field [69]. The same force field has been used by other MD studies on the SARS-CoV-2 Spike protein [70], [71]. Sialic acid topology was computed using the SwissParam server [72]. Proteins were placed inside a dodecahedric simulative box filled with TIP3P water molecules [73]. We ensure that the gap between the box and the outermost atom is at least 10 Å in all simulated systems. If necessary, sodium or chloride ions were added to neutralise the total charge of the system. The steepest descent algorithm up to 50,000 steps was performed to minimise the system. We performed 100 ps NVT and NPT steps at a 2 fs time-step. The temperature was kept constant at 300 K using a v-rescale thermostat [74], and the ultimate pressure was set to 1 bar using a Parrinello-Rahman barostat [75]. The LINCS algorithm [76] was used to constrain bonds involving hydrogen atoms. The short-range non-bonded interactions were evaluated using a cut-off of 12 Å. and the long-range electrostatic interactions using the Particle Mesh Ewald method [77]. The molecular dynamics time step was of 2-fs and all the simulations were run for 40 ns. For every system, we performed the simulations thrice.

## 3 Results

### 3.1 Phylogenetic studies of coronavirus NTD domains

**Fig. 1:**
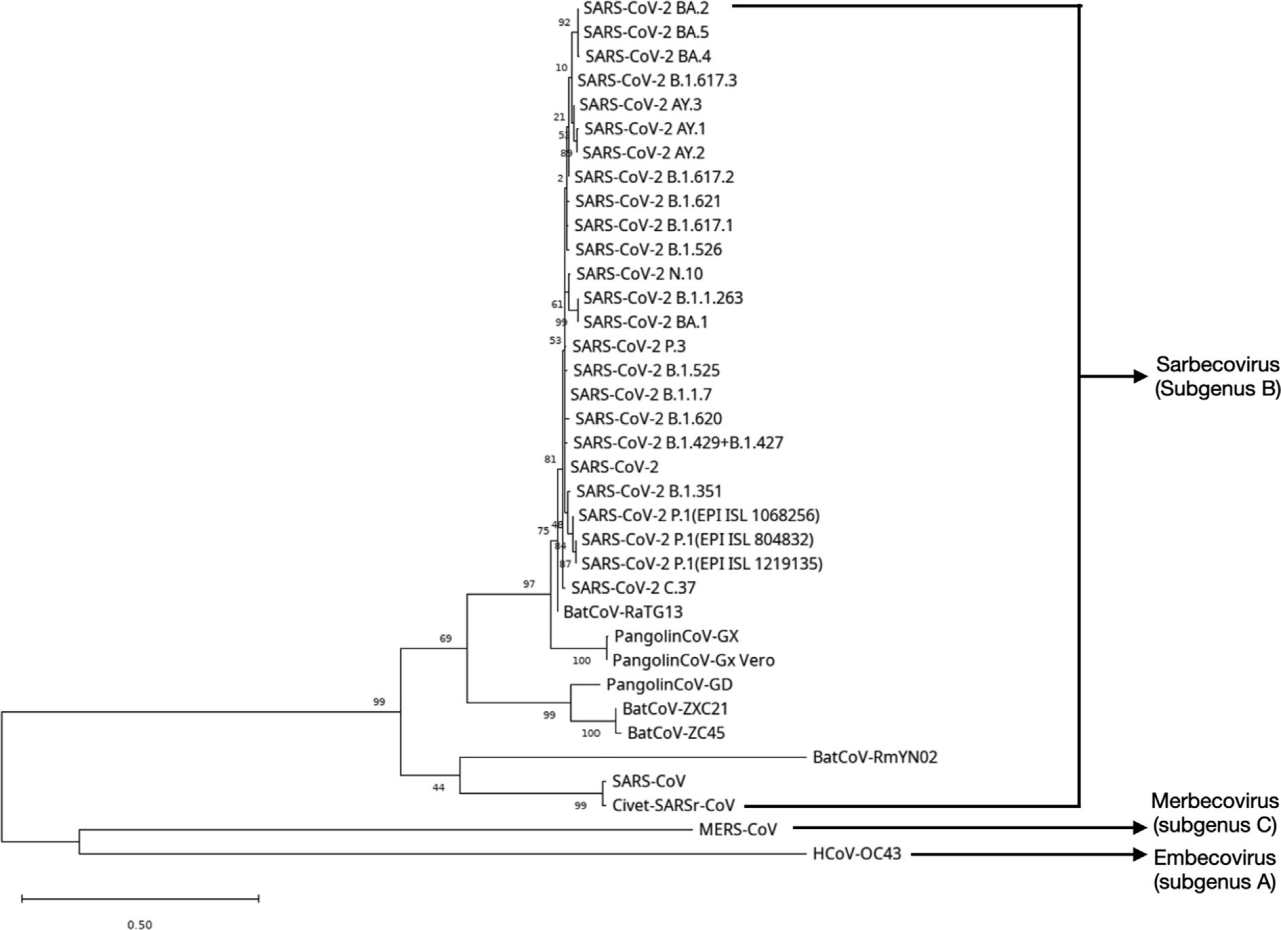
Phylogenetic tree of BCoV NTD amino acid sequences. The phylogenetic tree was inferred according to the Maximum Likelihood method. Genetic distance was computed using the Whelan and Goldman model and gamma-distributed rate variation among sites (WAG + G). SARS-CoV-2, BatCOV-RaTG13, PangolinCoV and BatCoV-ZXC21 and BatCoV-ZC45 fall into the same clade in the tree. In contrast, SARS-CoV, Civet-SARSr-CoV and BatCoV-RmYN02 were found to belong to another clade. HCoV-OC43, which is thought to have emerged in the 1950s [79], [80] and MERS-CoV are more distant from the others. See Supplementary Fig. 1 for a bootstrap tree.

We performed phylogenetic studies on the NTD domain to explore the relationships between the different BCoVs. We obtained sequences of the NTD domain for 36 BCoVs from the NCBI GenBank database and GISAID database. BCoVs have been classified into 5 subgenera: Embecovirus (subgenus A), Sarbecovirus (subgenus B), Merbecovirus (subgenus C), Nobecovirus (subgenus D) and Hibecovirus [37], [78]. SARS-CoV-2 and SARS-related coronaviruses belong to the Sarbecovirus subgenus (subgenus B). HCoV-OC43 belongs to Embecovirus (subgenus A), while MERS-CoV was classified into Merbecovirus (subgenus C). So far, there have been no reports of human-infecting BCoVs from other subgenera. Fig. 1 demonstrates that the phylogenetic tree of SARS-CoV-2 based on the NTD domain is similar to those constructed from other parts of the Spike protein.

### 3.2 Structural classification of NTD domains from BCOVs and analysis of pockets in the NTD domain

**Fig. 2:**
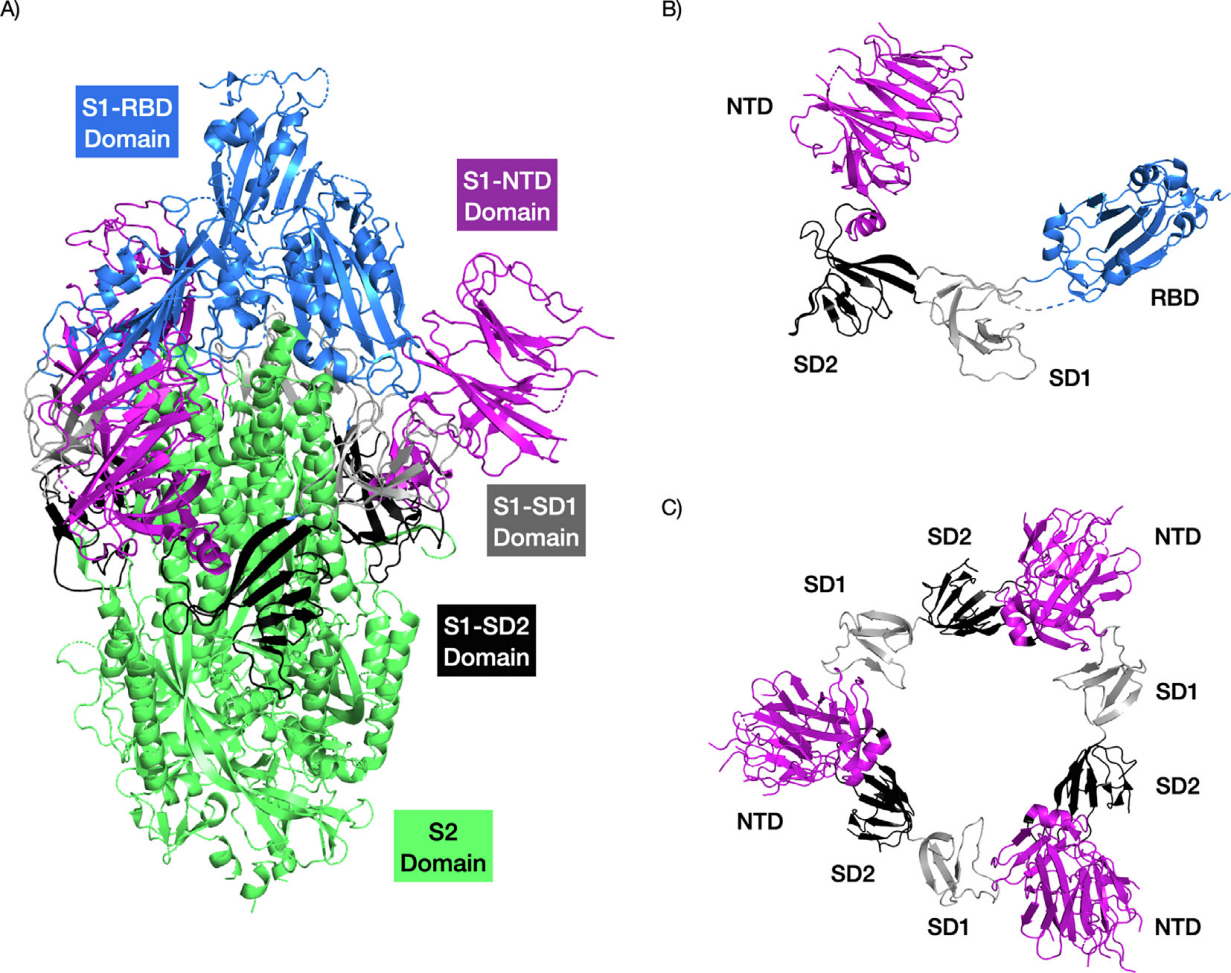
Structure of the SARS-CoV2 Spike protein highlighting the different domains. The RBD domain is coloured in blue, the NTD domain in purple, the SD1 domain in grey and the SD2 domain in black. The trimer complex is shown in (A). The S1 region of a Spike protein monomer is shown in (B). The interactions of the NTDs with SD1 and SD2 domains of the Spike protein trimer are shown in (C). PDB structure 6VSB. (For interpretation of the references to colour in this figure legend, the reader is referred to the web version of this article.)

The structures of the Spike proteins from the Wuhan-1 strain of SARS-CoV-2 and several other strains (i.e. SARS-CoV, PangolinCoV-GX, PangolinCoV-GD, BatCov-RaTG13) have been determined by experimental methods [81], [82], [83], [84]. The NTD domain interacts with a number of other domains within the Spike protein (subdomains SD1 and SD2, which serve as a hinge for RBD up-movement [85], [86]), however a significant proportion of the domain is surface accessible and able to interact with other compounds and proteins on the host cell surface (see Fig. 2). Structure analyses of the 4 BCoVs that are in the same subgenus (B) (see above) as SARS-CoV-2, showed that the NTD domains are all assigned to the same evolutionary superfamily (ID:2.60.120.960) in the in-house CATH domain structure classification [19]. This superfamily falls in the fold group (2.60.120) containing the human sugar-binding protein Galectin-3.

**Fig. 3:**
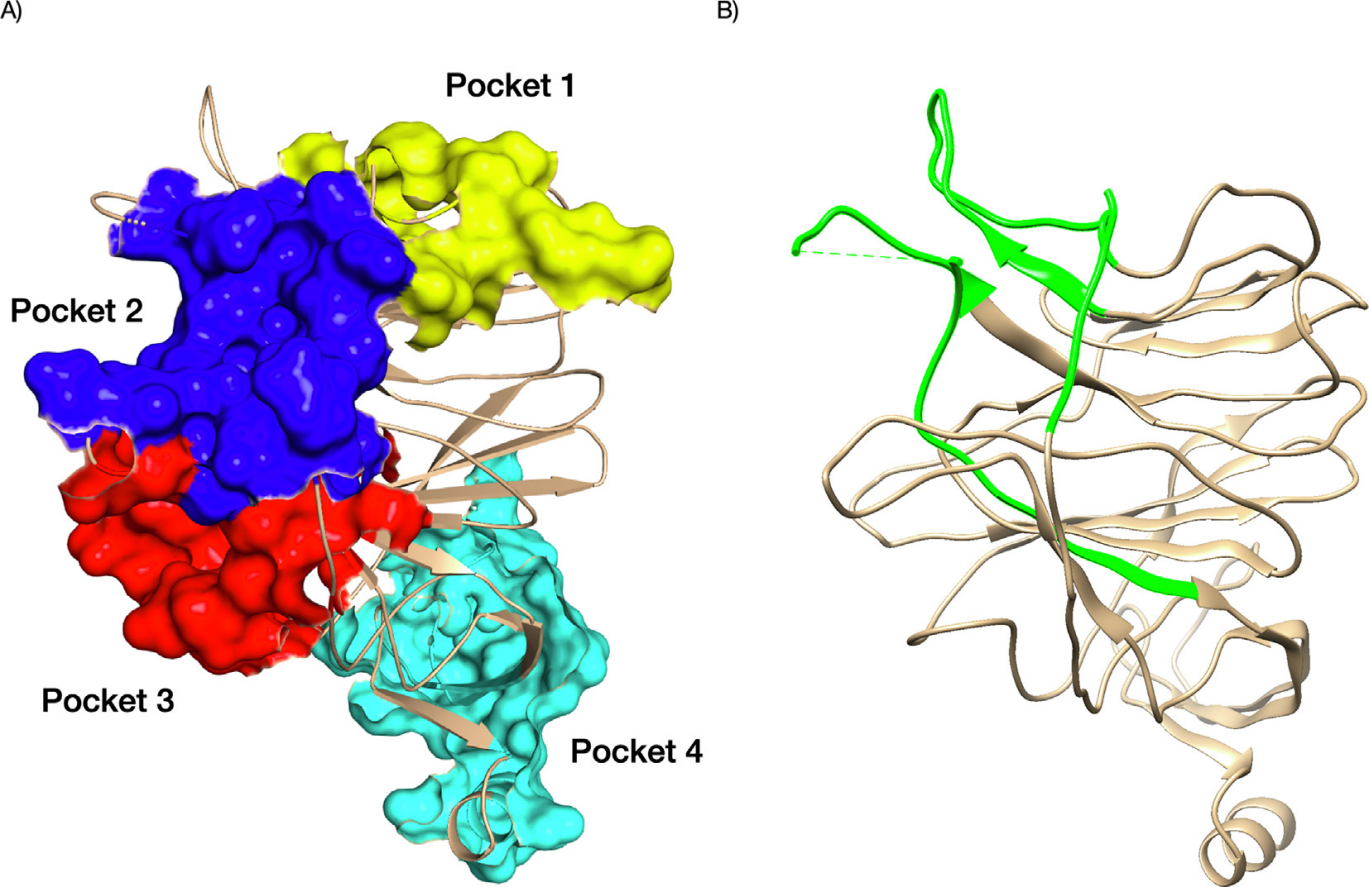
Pockets identified in NTD domain of SARS-CoV-2 (A) and antigenic NTD supersite of SARS-CoV-2 is shown in green (B). For (A), we coloured Pocket 1 in yellow, Pocket 2 in blue, Pocket 3 in red and Pocket 4 in cyan. PDB structures 7C2L. (For interpretation of the references to colour in this figure legend, the reader is referred to the web version of this article.)

As mentioned above, there is evidence for at least three pockets in SARS-CoV-2 [22], [29], [31], [33], [35], which are known (Pocket 2) or predicted (Pockets 1, 3) to bind sialic acid/sugars. To confirm and further characterise these and search for additional pockets we used cavity detection and docking tools. Analysing the structure of the SARS-CoV-2 NTD domain using CavityPlus [61], we identified the known sugar binding pocket shared with Galectin-3 (Pocket 1, Fig. 3). Two other cavities were confirmed (Pockets 2 (blue) and 3 (red) in Fig. 3) that share, through a common loop, a reported conserved sugar-binding motif (comprising 7 residues - G72, T73, N74, G75, T76, K77 and R78). As mentioned above Pocket 2 of SARS-CoV-2 has been experimentally demonstrated to bind sialic acid [35]. Furthermore, in agreement with McCallum et al. (2020), we observe that the antigenic NTD supersite coincides with sugar-binding pockets 1 and 2 [18]. Finally, a fourth, previously undefined pocket was detected (Pocket 4, colour cyan) shown in Fig. 3 below (See Section 3.8 for more details). Supplementary Table 4 shows the SARS-CoV-2 NTD residues that form the pockets detected by our study.

### 3.3 Analysis of structural similarity between the coronavirus NTD domains

**Fig. 4:**
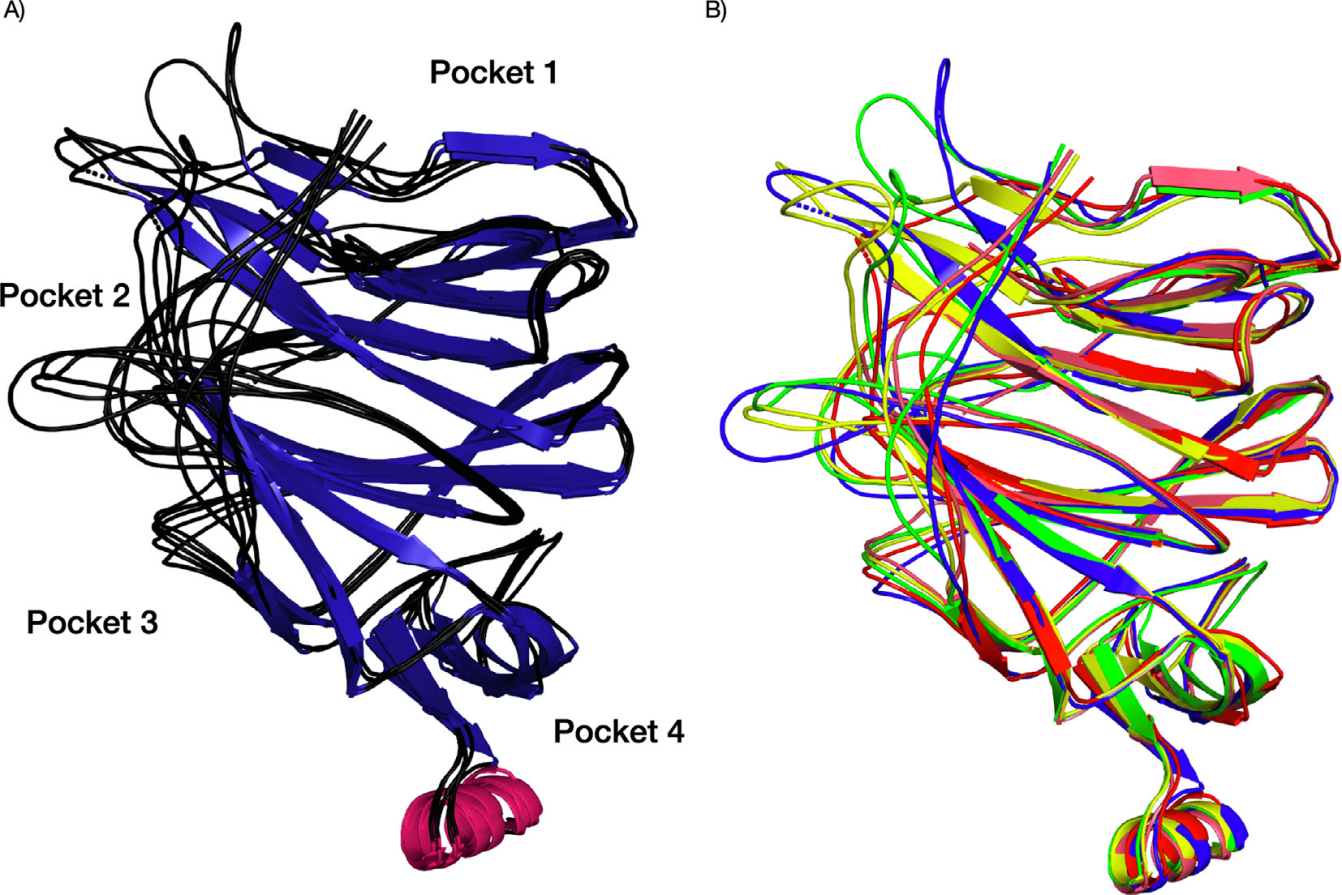
Structure superposition of CoV NTDs (SARS-CoV, PDB ID 6ACC, Pangolin CoV GX, PDB ID 7CN8, Pangolin-CoV-GD, PDB ID 7BBH, Bat-Cov-RaTG13, PDB ID: 7CN4, SARS-CoV-2, PDB ID 7C2L). The structures are coloured based on their secondary structure components (A) and proteins (different species/variants – SARS-CoV (red), Pangolin CoV GX (yellow), Pangolin-CoV-GD (orange), Bat-Cov-RaTG13 (green), SARS-CoV-2 (blue)) (B). (For interpretation of the references to colour in this figure legend, the reader is referred to the web version of this article.)

To determine how conserved this domain is across these 4 BCoVs in the same lineage (B) (see above) as SARS-CoV-2, and identify any variable regions, we performed a structure superposition using our in-house protein structure alignment tool SSAP (see Materials and methods). Overall, the structures are very similar (see Fig. 4) with an average SSAP of 86 (range is 0 to 100) and RMSD value of 2.16 Å suggesting a high global conservation of this domain across these BCoVs. However, whilst a large core of the domain is highly conserved, there are extensions in some of the loops corresponding to the known and putative sugar binding pockets. The variable nature of these loop regions across multiple BCoV NTD domains had previously been observed via a multiple sequence alignment [17], but the authors did not perform structural analyses to assess their proximity to putative sugar-binding regions. However, as mentioned above, there is evidence in other BCoVs that these changes may improve affinity for sugars on the host cell surface and therefore increase the affinity of the virus for the specific host cell [24], [25].

### 3.4 Comparison of the multiple sequence alignment of the BCoVs with structural data reveals evolutionary hotspots near known and predicted sugar binding pockets

**Fig. 5:**
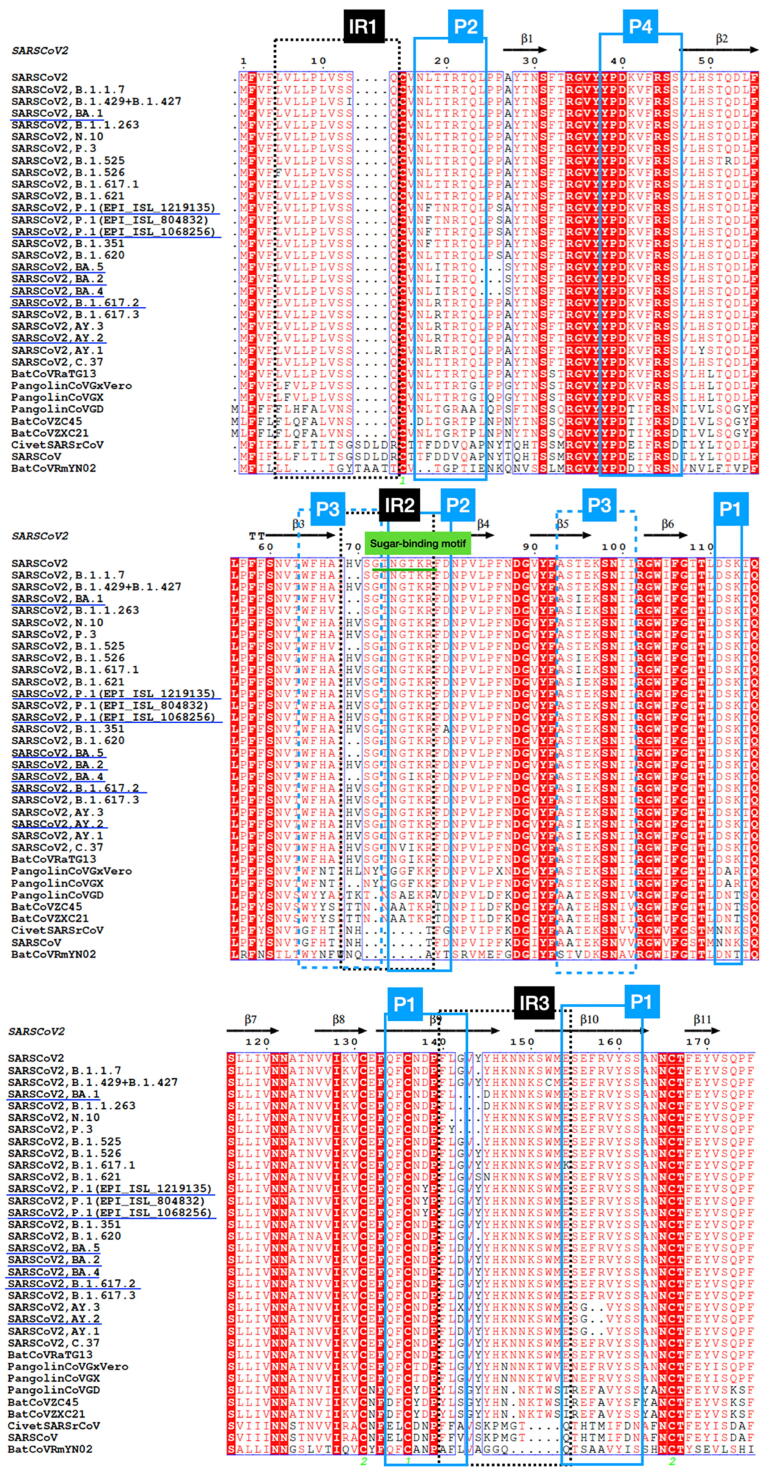

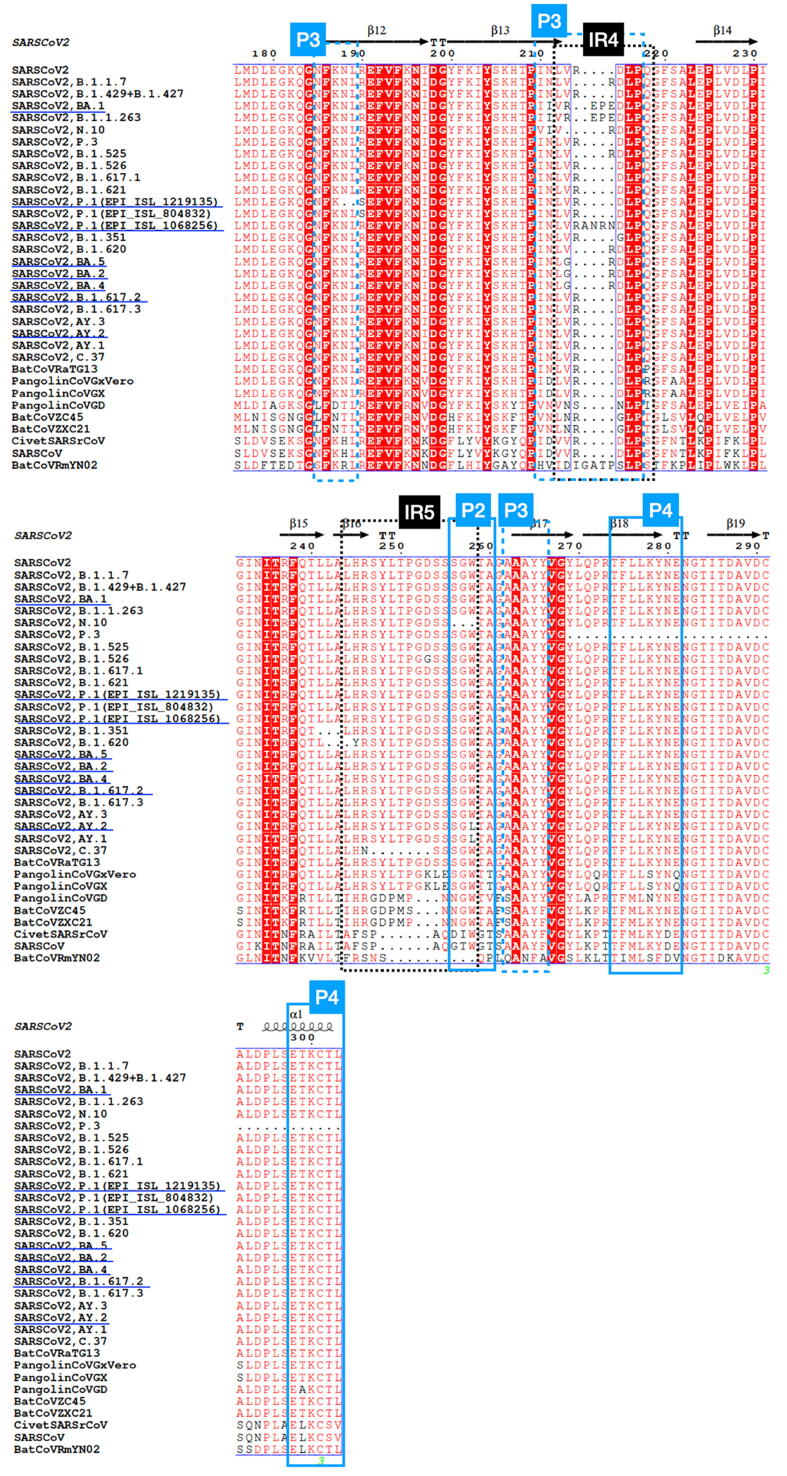
Multiple sequence alignment of BCoV NTDs. The Indel region (IRs), sugar-binding motif and known/putative sugar-binding pockets (P) are highlighted. Indel regions (IR1-IR5), pockets (P1- P4). VOCs having variants that affect binding energy are underlined (i.e., P.1 Gamma variant, B.1.617.2 Delta variant, AY.2 Delta Plus variant, BA.1 Omicron variant, BA.2 Omicron variant, BA.4 Omicron variant, BA.5 Omicron variant).

Our structural analyses suggested high structural variability of Pockets 1, 2 and 3 in BCoVs. We extended this analysis by performing a sequence-based analysis of a much larger set of BCoVs. Garry et al. (2021) had previously shown the value of using a multiple sequence analysis of multiple BCoVs and strains of SARS-CoV-2 to highlight regions varying across the BCoVs [17]. We revisited this on a larger set of 34 BCoVs (including only Sarbecovirus subgenus sequences thus most of our sequences are SARS-CoV-2 variants, and there is an evolutionary imbalance in terms of sequence sampling), exploiting our structural data to produce a structure-based multiple sequence alignment (MSA), using ESPript, since structural data typically gives a more accurate alignment (Fig. 5). We used our structural data to reveal the nature of highly variable positions in the alignment. In support of previous studies, we observe that lineage indels are a common feature of the Spike protein in BCoVs, with seven major loops most frequently affected. Five out of seven loops involve the NTD. Since we had structurally characterised the binding pockets in SARS-CoV-2, we mapped these pocket regions onto the MSA and analysed the sequence variability in those regions.

Whilst residues in most regions of the protein are highly conserved (ScoreCons values >80), the residues contributing to putative sugar binding Pocket 1 are somewhat less conserved (average ScoreCons of 70) and even more so for Pocket 2 (average ScoreCons of 61). Pocket 2 has changed in some BCoVs (SARS-CoV, civet SARS-CoV and BatCoV-RmYN02). Cheng et al. (2019) also compared BCoVs NTD structures and found Pocket 2 of SARS-CoV to be less well-defined with the loops not giving such a well-formed pocket [27]. Our docking studies (see Section 3.6 below) suggest that Pocket 3 is also able to bind sialic acid/sugars. This pocket is also quite variable with average ScoreCons values of 73. However, Pocket 4 is very conserved relative to the other pockets with an average ScoreCons value of 87.

**Fig. 6:**
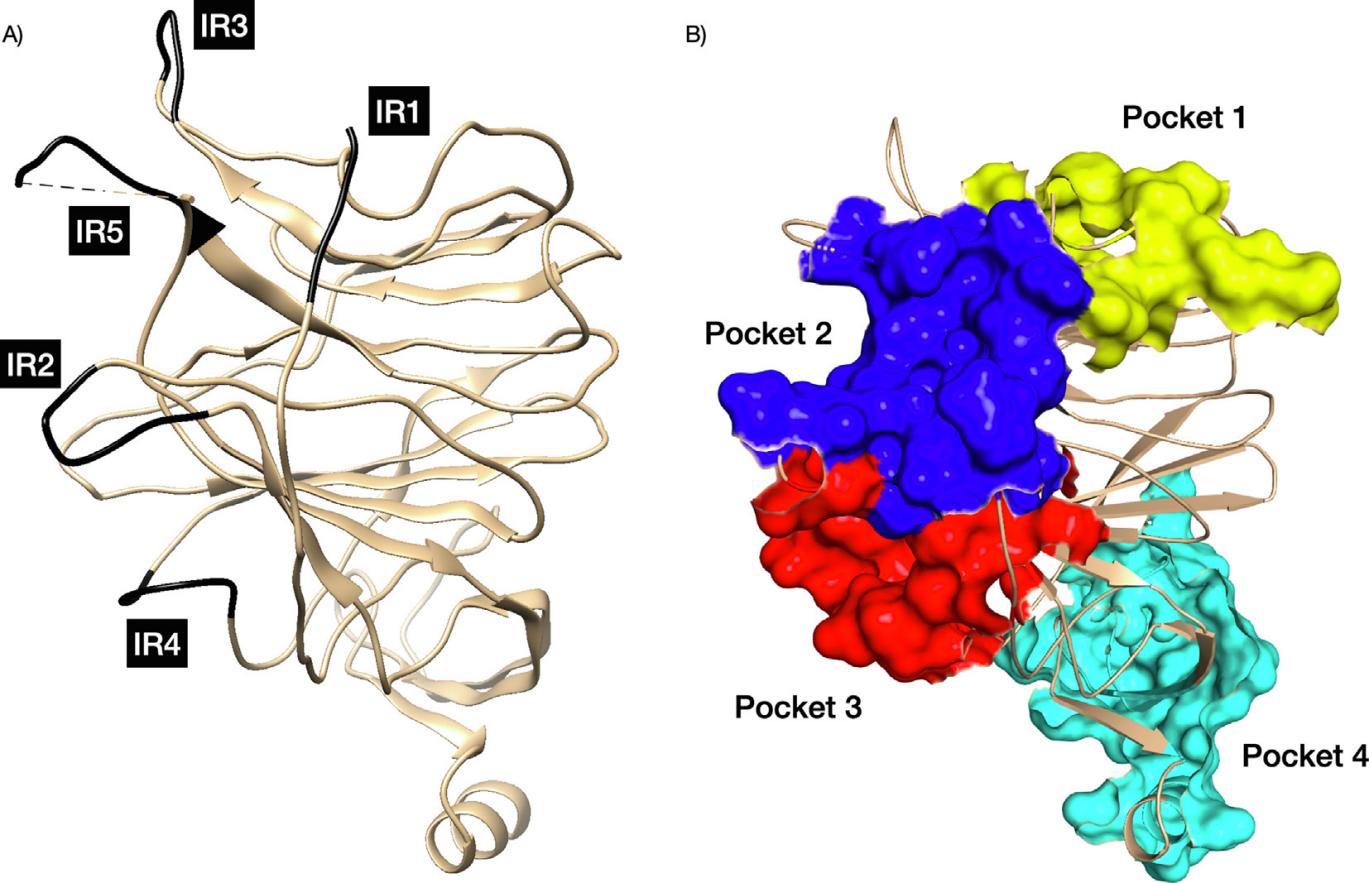
Indel regions and binding pocket of SARS-CoV-2 NTD. (A) Indel regions (IR) were identified using the MSA. (B) Highlighting the sugar-binding pockets, we coloured Pocket 1 in yellow, Pocket 2 in blue, Pocket 3 in red and Pocket 4 in cyan. PDB structure 7C2L. (For interpretation of the references to colour in this figure legend, the reader is referred to the web version of this article.)

The proximity of the indel regions to residues in these known and putative sugar-binding pockets is shown in Table 1, below. Most indels are very close to Pockets 2 and 3 (i.e., <2 Å). Furthermore, indel region 2 (close to Pockets 2 and 3), and indel region 4 (close to Pocket 3) and 5 (close to Pocket 2) are the most variable regions across these BCoVs. This suggests that these binding pockets are hotspots for viral evolution (Fig. 6). Indeed, most of the indels occur on this side of the NTD domain (See Fig. 6) and seem to target the sugar-binding pockets, possibly tuning the interactions with sugars such as sialic acids.

**Table 1:** Proximity of indel regions to residues in the sugar binding pockets. If the indel region lies close to multiple pockets (<5Å), minimal residue distances to all pockets are provided.

Indel region	Minimal residue distance to known/putative sugar binding pockets (Å)
Indel region 1	1.86 (Pocket 1) 1.33 (Pocket 2)
Indel region 2	1.33 (Pocket 2) 1.33 (Pocket 3)
Indel region 4	1.31 (Pocket 3)
Indel region 5	1.31 (Pocket 2)

### 3.5 Structural analysis of a broad set of BCOVs to better understand the evolution of the sugar-binding pockets

Building on the sequence analysis, we revisited our structure comparison of BCOVs (Section 3.3 above) and analysed further the structural features observed in a larger set of BCoV domains in the CATH superfamily containing NTD domains (see Supplementary Fig. 2). As suggested from the MSA, the loops around Pocket 2 seem to be highly structurally variable. This pocket is less well defined in SARS-CoV and comparing the structures and sequences of HCoV-OC43 and SARS-CoV-2 we see strong innovation in Pocket 2 in SARS-CoV-2 suggesting alterations in this pocket were important for the evolution of this virus. Similarly, structural analyses comparing Pocket 3 across the coronaviruses suggests that this pocket can be highly variable too, and may therefore offer another potential sialic acid binding site. We used docking studies (see below) to explore and contrast the ability of the different pockets in SARS-CoV-2 to bind sialic acid.

### 3.6 Docking analyses of sialic acid in pockets of SARS-CoV-2

Various computational studies had indicated that Pocket 1, which is similar to the experimentally characterised sugar binding pocket in Galactin-3, could bind sugar [22], [28], [31]. Furthermore, previous *in-silico* docking studies showed that sialic acids could bind to Pocket 2 [87]. More compelling, recent experimental studies confirm sialic acid binding to Pocket 2 [35]. We used computational analyses to explore and contrast the binding of sialic acid to all pockets in the NTD domain.

**Fig. 7:**
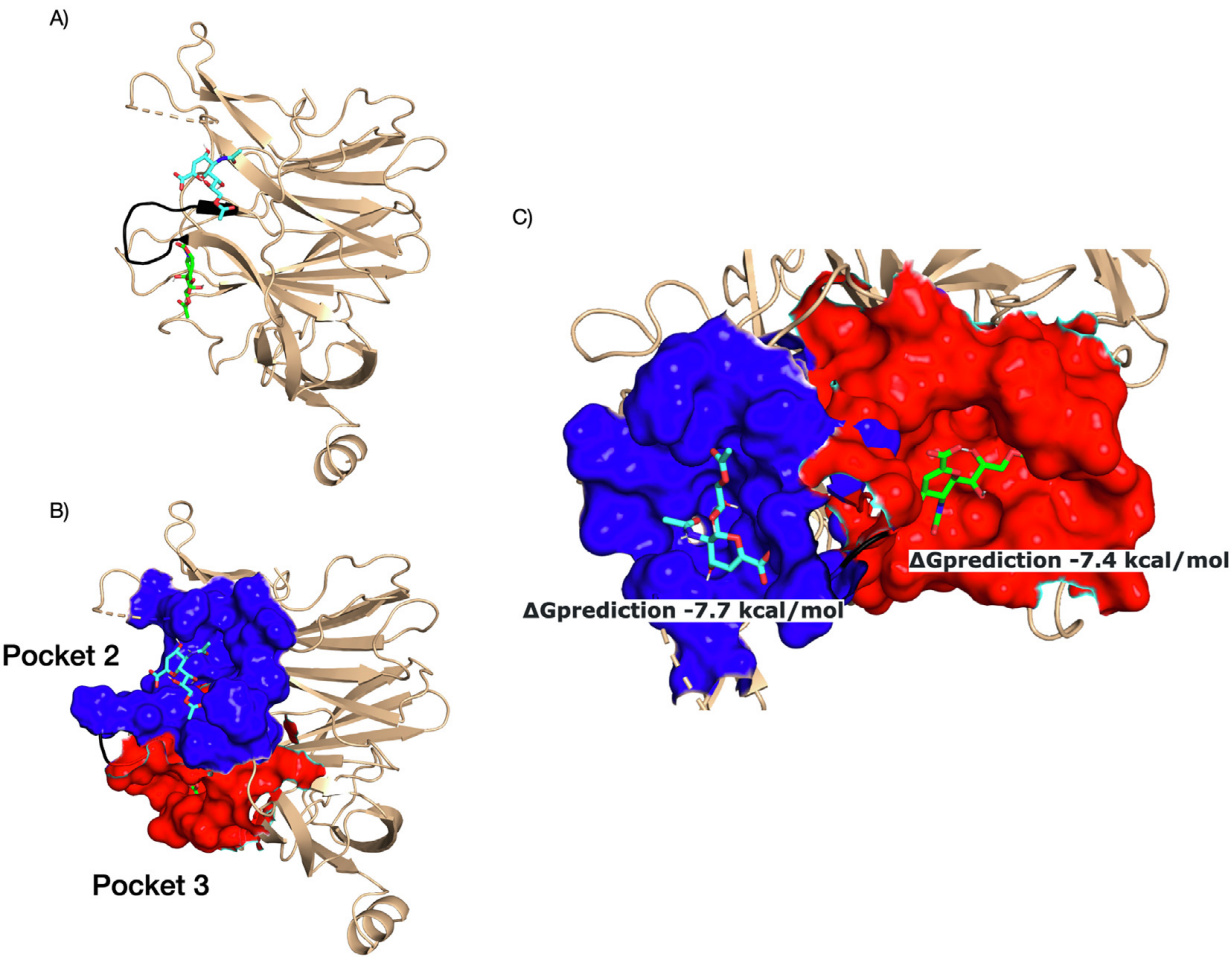
Sialic acid docked into two cavities in the SARS-CoV2 NTD domain. A) the sugar-binding motif is shown in black, following the same orientation as in the other figures. B) cavities were identified by CavityPlus and are shown using surface representation and coloured blue (Pocket 2) and red (Pocket 3) respectively. C) the structure is oriented to show both cavities and we give the PRODIGY predicted binding energy. (For interpretation of the references to colour in this figure legend, the reader is referred to the web version of this article.)

We first determined the binding energy of sialic acid bound to Pocket 2 to a beta-coronavirus HCoV-OC43 for which a structure had been solved with sialic acid bound in Pocket 2 [30], using the PRODIGY score of HADDOCK and obtained a value of -7.1 kcal/mol. This was done to determine the threshold level of binding energy for which there is experimental data confirming binding. We then used HADDOCK to dock sialic acid into all four of the pockets in the experimental structure of the NTD domain of SARS-CoV-2, and calculated the PRODIGY predicted binding energy of sialic acid (See Fig. 7). For Pocket 2 and 3 we observe comparable predicted binding energy to that predicted for Pocket 2 of HCoV-OC43 for which there is experimental confirmation of binding [24]. Our results show that sialic acid binds to both Pocket 2 (-7.7 kcal/mol), for which recent experimental confirmation has been reported [35], and Pocket 3 (-7.4 kcal/mol) (see Table 2).

**Table 2:** PRODIGY predicted binding energy of sialic acid to NTD pockets of SARS-CoV and SARS-CoV-2.

Virus	PRODIGY predicted binding energy (kcal/mol)			
	Pocket 1	Pocket 2	Pocket 3	Pocket 4
SARS-CoV	−5.5	−6.4	−6.6	−6.4
SARS-CoV-2	−6.6	−7.7	−7.4	−6.6

Since Pockets 2 and 3 show the most variability across BCoVs and bind sialic acid more strongly than the other pockets we subsequently used LigPlot+ to further analyse the interactions of sialic acid with residues in these pockets. For Pocket 2 (see Supplementary Fig. 3), 3 residues of the sugar binding motif (T76, K77 and R78) are involved in binding with sialic acid. K77 forms a hydrogen bond and hydrophobic interaction with sialic acid, while T76 and R78 form hydrophobic interactions with the compound. For Pocket 3, there are no polar interactions of the ligand with residues in the sialic acid binding pocket. However, we do observe hydrogen bonds and van der Waals interactions with sialic acid. Further, analyses of these pockets for other BCoVs and also for Pockets 1 and 4 showed much weaker interactions (see Supplementary Table 5). We also explored the effects of *in-silico* mutagenesis on sialic acid binding in Pockets 2 and 3. There were no impacts for Pocket 3 (see Supplementary Table 7) but in Pocket 2 binding was significantly reduced for two of the binding residues (i.e., R78 and T259, see Supplementary Table 6).

In summary, our structural, sequence and sialic acid binding analyses suggested that Pockets 2 and 3 which are more highly varying in their sequence and structure properties across BCoVs, also show stronger binding with sialic acids.

### 3.7 Analysis of recent variants of concern/interest in SARS-CoV-2

In order to assess the possible impact of recent variants of concern/interest in relation to these sugar binding pockets, we assessed the impacts on the binding for the various mutations in the VOCs and VOIs. We did not consider Pocket 4 as this is highly conserved across BCoVs and no VOC variants occurred in this pocket. We list all the NTD mutations/insertions found in the different VOCs/VOIs in Supplementary Table 8. Many of the mutations/insertions lie close to the 3 known and putative sugar binding pockets discussed in this and related studies.

We report the PRODIGY binding energy for the individual NTD mutations in Supplementary Table 9. Overall, most of the mutations in the VOCs and VOIs were not predicted to drastically alter or enhance sialic acid binding. However, three mutations, one observed in Pocket 1: E154K (found in Kappa variant) and the other two observed in Pocket 3: T95I (found in Kappa, Delta, Delta Plus (AY.1, AY.2) Iota, Mu and B.1.1.263 variants) and V213G (found in Omicron (BA.2, BA.4 and BA.5)) were predicted to increase binding affinity (≥0.5 kcal/mol changes), as compared to the original Wuhan-1 strain of SARS-CoV-2. See Supplementary Fig. 4 and Supplementary Fig. 5 for LigPlots.

We also examined the impact on binding energy when all the mutations and indels in the strain were considered simultaneously (see Supplementary Table 10). For Pocket 1, the binding of the B.1.617.2 Delta strain, AY.2 Delta plus strain, two of the Gamma P.1 strains, BA.2 Omicron strain, BA.4 Omicron strain and BA.5 Omicron strain to sialic acid are stronger than the original Wuhan-1 strain (an increase of ≥0.5 kcal/mol, 2.33-fold increase of binding constant K_b_). It can be seen that there is an increase from 3 hydrogen bonds in the Wuhan-1 strain to 4 hydrogen bonds in the Omicron BA.4 variant with binding energy increase of 0.7 kcal/mol (i.e., 3.28-fold increase of binding constant K_b_) (See Supplementary Fig. 7). For Pocket 2, there is no significant difference among the SARS-CoV-2 strains. But, we do see an increase from 4 hydrogen bonds in the Wuhan-1 strain to 7 hydrogen bonds in the Iota variant (See Supplementary Fig. 7).

However, for Pocket 3 we see a stronger binding energy (an increase of >0.5 kcal/mol, 2.32-fold increase of binding constant K_b_), compared to the original SARS-CoV-2, in the following strains of SARS-CoV2: B.1.617.3, B.1.429 + B.1.427 (Epsilon variant), B.1.526 (Iota variant), C.37 (Lambda variant), P.3 (Theta variant), N.10, B.1.1.263, BA.1 (Omicron variant) and BA.2 (Omicron variant). There is an increase from 3 hydrogen bonds in the Wuhan-1 strain to 7 hydrogen bonds in the Omicron BA.1 variant with binding energy increase of 1 kcal/mol (i.e., 5.41-fold increase of binding constant K_b_) (See Supplementary Fig. 7 and Supplementary Table 10). However, it is important to note that these predictions are to some extent an approximation and need to be verified by careful experimental work to confirm these effects.

To further explore the impacts of variants, we performed molecular dynamics simulations using the original Wuhan-1 strain and the strain that shows the highest deviation from the Wuhan-1 strain for each pocket (Pocket 1: SARS-CoV-2 wild Wuhan-1 versus SARS-CoV-2, BA.4, Omicron variant; Pocket 2: SARS-CoV-2 wild Wuhan-1 versus SARS-CoV-2, B.1.526, Iota variant; Pocket 3: SARS-CoV-2 wild Wuhan-1 versus SARS-CoV-2, B.1.1.263 and BA.1, Omicron variant).

Our MD analysis demonstrates that sialic acid can interact with all three pockets in all VOC/VOIs analysed. In all 3 pockets, strains with the highest deviation from the original Wuhan-1 strain remain bound for at least 19 ns (See Table 3). Sialic acid was also observed for a longer period (up to the whole simulated time of 40 ns) in these selected strains compared to the original Wuhan-1 strain. We then investigated the number of hydrogen bonds formed between NTD and sialic acid of each system. Overall, we can see recent variants form more hydrogen bonds than the original Wuhan-1 strain across all three pockets (See Table 3).

**Table 3:** Time permanence (in ns) of sialic acid bound to NTD pocket and average number of hydrogen bonds formed between sialic acid and SARS-CoV-2 wild Wuhan-1 strain/the strain that shows the highest deviation for each pocket.

Pocket	Strain	Permanence of sialic acid bound/Average number of hydrogen bonds formed		
		1st MD run	2nd MD run	3rd MD run
1	Wuhan-1	28.38 ns/1.19	1.07 ns/1.01	22.64 ns/1.19
	Omicron BA.4	23.36 ns/2.81	19.53 ns/1.94	40.00 ns/2.07
2	Wuhan-1	40.00 ns/3.77	22.19 ns/3.22	22.81 ns/4.34
	Iota B.1.526	40.00 ns/4.55	40.00 ns/6.12	40.00 ns/7.79
3	Wuhan-1	18.72 ns/2.07	19.97 ns/2.85	8.62 ns/2.51
	Omicron BA.1 and B.1.1.263	40.00 ns/3.66	22.67 ns/3.55	40.00 ns/3.71

**Fig. 8:**
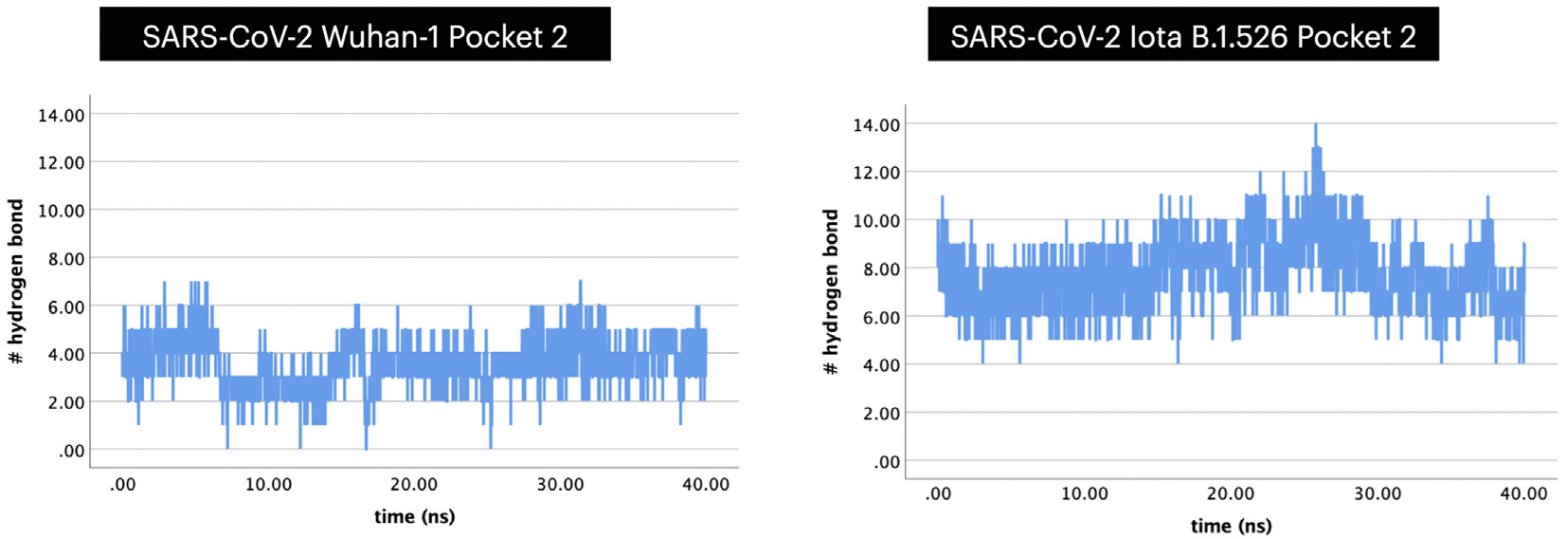
Time evolution of the number of hydrogen bonds formed between the original Wuhan-1 strain and the strain (i.e., Iota B.1.526) that shows highest deviation to sialic acid for Pocket 2.

For Pocket 1, the Omicron BA.4 strain forms more hydrogen bonds (average 2.25, max 6) than the original Wuhan-1 strain (average 1.19, max 5). For Pocket 2, the Iota B.1.526 strain forms more hydrogen bonds (average 6.15, max 14) than the original Wuhan-1 strain (average 3.78, max 10). We illustrate the best MD run between Pocket 2 of SARS-CoV-2 NTD and the strain that shows highest deviation (i.e., Iota B.1.526 strain) to sialic acid in Fig. 8. For Pocket 3, the Omicron BA.1 strain forms more hydrogen bonds (average 3.65, max 9) than the original Wuhan-1 strain (average 2.48, max 7). Our results also show that sialic acid binds more strongly to Pockets 2 and 3 compared to Pocket 1. See Supplementary Figs. 7 and 8 for plots of ligand RMSD and number of hydrogen bonds for all the 3 runs for all 3 pockets.

It is also important to consider how these effects compare with impacts of mutations in the RBD domain on binding to ACE2 in the host cell. Our computational analyses of RBD mutations in the VOCs (using mCSM-PPI2 [60], see Materials and methods), found that only one mutation in the RBD domain, the S477N mutation (ΔΔG of 0.57 kcal/mol) of the Omicron variant enhances binding to the ACE2 receptor (See Supplementary Table 11). The degree of impact is comparable to that measured for the increase in affinity of the NTD complex with sialic acid in Pocket 3 for the Omicron variant (1.0 kcal/mol).

### 3.8 Pockets 1,2 and 3 are variable across BCoVs but structural analyses reveal a fourth pocket that is highly conserved and potentially druggable

**Fig. 9:**
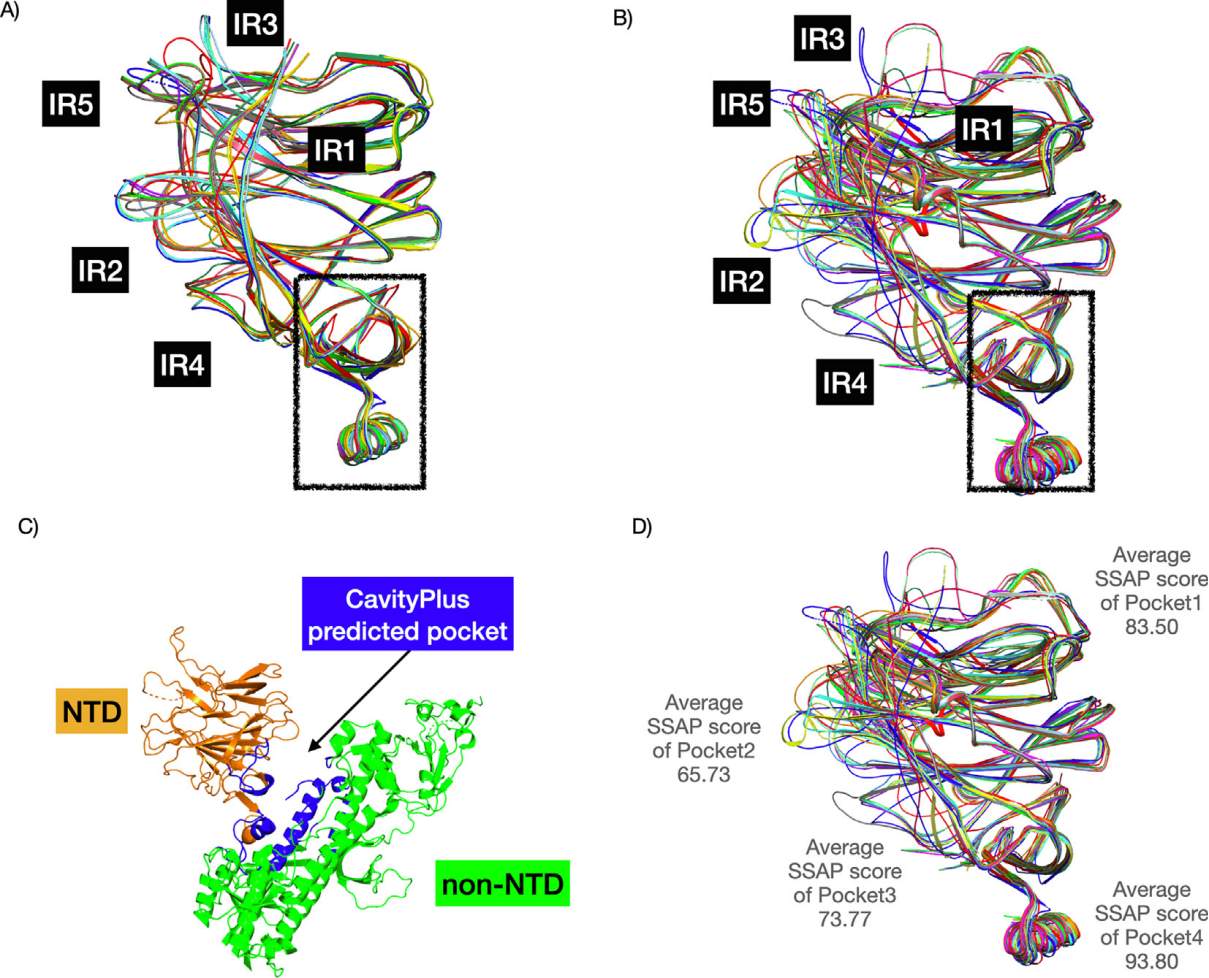
Structure superposition of CoV NTDs. A) comprises the 34 BCoV NTDs in the multiple sequence alignment (see Fig. 5). B) constitutes 24 BCoV NTDs from the CATH family. For A) and B), we used known structures and structural models (built using AlphaFold2). We found a highly conserved pocket (highlighted by the box) (score of 1838 - highly positive DrugScore constitutes high druggability) that could constitute a good pan coronavirus drug target. C) demonstrates this pocket predicted by CavityPlus in blue. D) We computed the structural conservation of pockets by calculating the average SSAP score. (For interpretation of the references to colour in this figure legend, the reader is referred to the web version of this article.)

To more fully explore the structural conservation of BCoVs, we expanded our previous analysis in Section 3.5 by modelling the structures of the 34 BCoV NTDs in our multiple sequence alignment (see Fig. 5) which had no experimental structure. We also modelled the structures of all BCoVs classified in the same superfamily in CATH (see Materials and methods Section 2.2). Only good quality models (predicted IDDT score >75, See Supplementary Table 3 for details) were used. Structural superpositions of the known (PDB) and modelled structures of BCoV NTD domains are shown in Fig. 9A. We assessed the conservation of the structures (see Fig. 9B). Overall, the conservation of the structures is high with an average SSAP of 87 (out of a maximum score of 100) and an RMSD value of 2.91. We also calculated the structure conservation for the pockets. Pocket 1 is conserved with an average SSAP of 83.50 (RMSD value of 5.77). There have been attempts to design drugs for this pocket and chloroquine and hydroxychloroquine have been tested and shown to bind in this pocket [22]. These drugs have been used for the treatment of malaria but are not being used for COVID-19. On the other hand, the structural conservation for Pockets 2 and 3 is low, with average SSAP scores of 65–73 (RMSD values of 8.39 and 5.59).

However, analysis by CavityPlus had identified a fourth druggable pocket (CavityPlus Drug Score of 1838, a highly positive DrugScore suggests high druggability), which also binds sialic acid but less strongly than the other pockets (See Fig. 9C). The structural conservation of this pocket is very high (average SSAP score of 93.80 and RMSD value of 2.26) (See Fig. 9D). The high structural conservation across BCoVs and the druggability of this pocket suggest that it could be a good pan coronavirus drug target.

To explore this pocket's conservation further, we generated another structure-based multiple sequence alignment of Pocket 4 and residues that are in its neighbourhoods, including more distant BCoVs (i.e. counting all BCoVs in the CATH superfamily, even relatives with low sequence identity <20% sequence identity). The alignment produced is informative with a diversity of positions (DOPs) score of 95.77. We found 10 highly conserved positions (ScoreCons value >0.70) within this druggable pocket, (See Supplementary Fig. 9 for more details). To test the significance of these highly conserved positions, we created 10,000 random models by randomly selecting 24 solvent-exposed positions (same residue number as Pocket 4) from the alignment and calculating these positions' average ScoreCons value. We found that the conservation of the Pocket 4 sialic acid binding residues was significantly different from random (p-value = 0, 1 sample *t*-test).

## 4 Discussion

Several experimental studies have reported that BCoVs use sialic acid-containing receptors for entry into the host cell [30], [88]. We have built on these studies and examined all known and putative binding pockets in the NTD domain of SARS-CoV-2 and related coronaviruses. Pockets 1, 2 and 3 had previously been identified and analysed by other studies suggesting sialic acid binding. We bring together this earlier data and our own data to strengthen these predictions [see Supplementary Table 12 summarising all evidences], and we add further information on the structural nature and evolution of these pockets in BCoVs generally. We also discover a fourth pocket not reported by other groups.

Recent experimental studies confirmed sialic acid binding to Pocket 2 [35]. Our docking studies found Pockets 2 and 3 to bind sialic acid more strongly than Pocket 1. Pocket 3 binds sialic acid as strongly as Pocket 2 and like Pocket 2 is highly variable across BCoVs. Unlike other studies on these pockets, our study also includes comprehensive analyses of Variants of Concern (VOCs) and Variants of Interest (VOIs) in these pockets. Our binding studies are further supported by MD simulations on the strain showing the highest deviation from the original Wuhan-1 strain. When considering all changes in the VOC, some significant changes in binding energy were linked to Pocket 3. Our main findings are discussed further below.

### 4.1 Pockets 2 and 3 are diversity hotspots and potential targets for the ongoing evolution of the virus and increased infectivity

Pockets 2 and 3 were found to be more variable, across the BCoVs than Pocket 1. Multiple lines of evidence including (1) the presence of a sugar binding motif [28], (2) NMR studies [34], [35], (3) Glyconanoparticle platform study [33], (4) detection of evolutionary hotspots, (5) impacts of residue deletions on infectivity [89], [90], (6) docking of sialic acid reported in [32], [35] and above (see Supplementary Table 12 for a summary of evidence) suggest a role in infection for these two pockets. Some of the changes we observed in these pockets across the beta coronaviruses could represent an ongoing adaptation of the virus to human cells with their specific sugar modifications. Whilst more evidence of sugar binding has accrued for Pocket 2, our studies of the impacts of VOCs in Pocket 3, summarised below, suggest that in monitoring changes in these pockets linked to transmission, consideration should be given to Pocket 3 as well as Pocket 2.

### 4.2 Differences in sugar-binding affinities across the variants of concern/interest in SARS-CoV-2 suggest a need for monitoring of Pocket 3

Recent SARS-CoV-2 variants show wide changes in infectivity [16]. We used established computational tools (HADDOCK and its scoring tool PRODIGY) and found that the largest increases in binding energies for the VOCs and VOIs were for Pocket 3 compared to the original Wuhan-1 strain of SARS-CoV-2 and SARS-CoV (see Supplementary Table 9). These energy changes are comparable in scale to changes in the binding of RBD to ACE2 (see Supplementary Table 10).

Notably, a recent study in Brazil [91] reported community transmission of different lineages of the variant of concern (VOC) gamma (P.1), which harbours NTD indels 69–70 in Pocket 3 and an insertion at 214, also in Pocket 3. These VOCs were thought to be responsible for the widespread transmission of SARS-CoV-2 in Brazil. In addition, the T95I mutation in Pocket 3 was reported to be one of the most prevalent mutations in the new (Delta Plus) strains of the Delta lineage (namely AY.2 or B.1.617.2.1) [89]. We found that combined mutations in Pocket 3 in VOCs/VOIs also cause changes in binding energy (B.1.617.3 (0.8 kcal/mol), B.1.429 + B.1.427 (Epsilon variant, 0.7 kcal/mol), B.1.526 (Iota variant, 0.8 kcal/mol), C.37 (Lambda variant, 0.8 kcal/mol), P.3 (Theta variant, 0.6 kcal/mol), N.10 (0.6 kcal/mol), B.1.1.263 (1.0 kcal/mol), BA.1 (Omicron variant, 1.0 kcal/mol) and BA.2 (Omicron variant, 0.9 kcal/mol). Our docking studies were further supported by MD simulations exploring impacts on sialic acid binding in the regions harbouring VOCs, as they demonstrated enhanced sialic acid binding to Pocket 2 for the VOCs and also showed enhanced sialic acid binding to Pocket 3 in the recent Omicron strain. Binding of sialic to Pocket 2 had previously only been demonstrated for the original Wuhan-1 strain [35].

Concern regarding the putative increase in transmission of the Omicron VOC highlights the need for further experimental characterisation of the roles of these NTD pockets in infection. In particular, the dramatic changes in virus entry efficiency achieved by changes to IR2 (near Pockets 2 and 3) should be studied further, especially by experimental studies to help in rationalising the likely impacts of emerging VOCs. We propose continuous monitoring of NTD mutations and indels (particularly for Pockets 2 and 3) in the context of newly emerging variants and their impacts on sugar-binding.

### 4.3 Predicted pockets: Implication in drug design

Dually targeting galectins and the sialic acid-binding domain of SARS-CoV-2 has been suggested as a promising strategy for COVID-19 for preventing viral entry and modulating the host immune response [90]. We observe high conservation in Pocket 1 (galectin-like binding pocket, structural similarity score of 83.50 out of 100) and Pocket 4 (score 93.80 out of 100). The druggable nature of Pocket 1 is also supported by the study by Fantini et al. [22] which showed that the drugs chloroquine and hydroxychloroquine fully mimic the way in which the sialic acid binds in Pocket 1 of NTD, and in the presence of these drugs SARS-CoV-2 no longer binds to sialic acid. However, neither of these compounds has been shown to be useful for treating COVID-19 and currently neither are being used for treatment of COVID-19.

In addition to Pocket 1, we suggest targeting Pocket 4, which is much more highly conserved among coronaviruses. Designing inhibitors that target structurally conserved regions or pockets in the BCoVs, such as the conserved binding pockets (1 and 4) found in SARS-CoV-2, seems to be a promising strategy to inhibit protein function and block viral entry [90]. Alternatively, the surface location and high structural conservation of Pocket 4 across BCoVs may also suggest a role for Pocket 4 in vaccine design.

## 5 Conclusion

In summary, our study brings together evidence from multiple computational and experimental investigations suggesting that NTD can bind sialic acid. Our structural analyses and docking studies agreed with previous studies suggesting a role for Pocket 2 in sugar binding. Binding to Pocket 2 has also been recently confirmed by an experimental study [35], thus validating our computational binding energy studies and MD simulations. In addition, our protocols were able to more deeply characterise the likely role of Pocket 3 in sugar binding. Whilst we predict the binding energy of this pocket to sialic acid to be slightly lower than for Pocket 2, we detected a residue mutation and indels in this pocket in recent VOCs/VOIs (e.g., the more recent Omicron strain) resulting in overall stronger binding energy for Pocket 3 compared to Pocket 2.

Since our analyses of Spike RBD domain, using the same strategies for measuring binding energy changes, showed no VOC/VOI mutations with significant impact on binding to ACE2 in the host cell, our detection of significant changes in sugar binding of NTD in these VOC/VOIs suggest a putative role for this pocket in aiding transmission.

We discovered a new pocket, Pocket 4, which we predict binds sialic acid less strongly than the other pockets, but which is more interesting because it has highly conserved structural and physicochemical properties that may make it a good potential target for vaccine and drug design.

## Declaration of Competing Interest

The authors declare that they have no known competing financial interests or personal relationships that could have appeared to influence the work reported in this paper.

## Appendix A Supplementary data

Supplementary data 1 The following are the Supplementary data to this article.
