# Supplementary material for: Structural and energetic analyses of SARS-CoV-2 N-terminal domain characterise sugar binding pockets and suggest putative impacts of variants on COVID-19 transmission
Source: Comput Struct Biotechnol J. 2022 Nov 7;20:6302–16. doi: 10.1016/j.csbj.2022.11.004 (PMC9639386; doi:10.1016/j.csbj.2022.11.004)
Supplement: Supplementary data 1 — The following are the Supplementary data to this article. [file mmc1.docx]

**Supplementary Material of**

**Structural and Energetic Analyses of SARS-CoV-2 N-Terminal Domain Characterise Sugar Binding Pockets and Suggest Putative Impacts of Variants on Covid-19 Transmission**


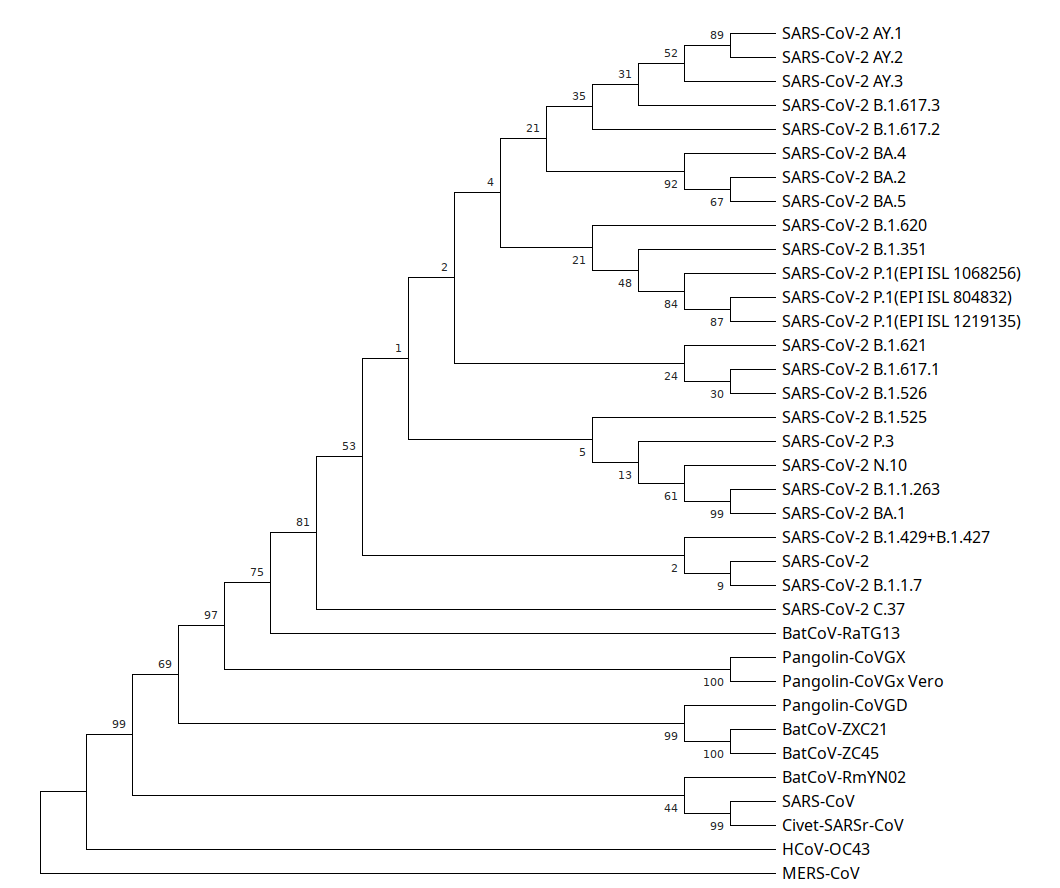


**Supplementary Figure 1.** Bootstrap consensus tree of BCoV NTD amino acid sequences. The phylogenetic tree was inferred according to the Maximum Likelihood method. Genetic distance was computed using the Whelan And Goldman model and gamma-distributed rate variation among sites (WAG + G). The bootstrap consensus tree was inferred from 1000 replicates.


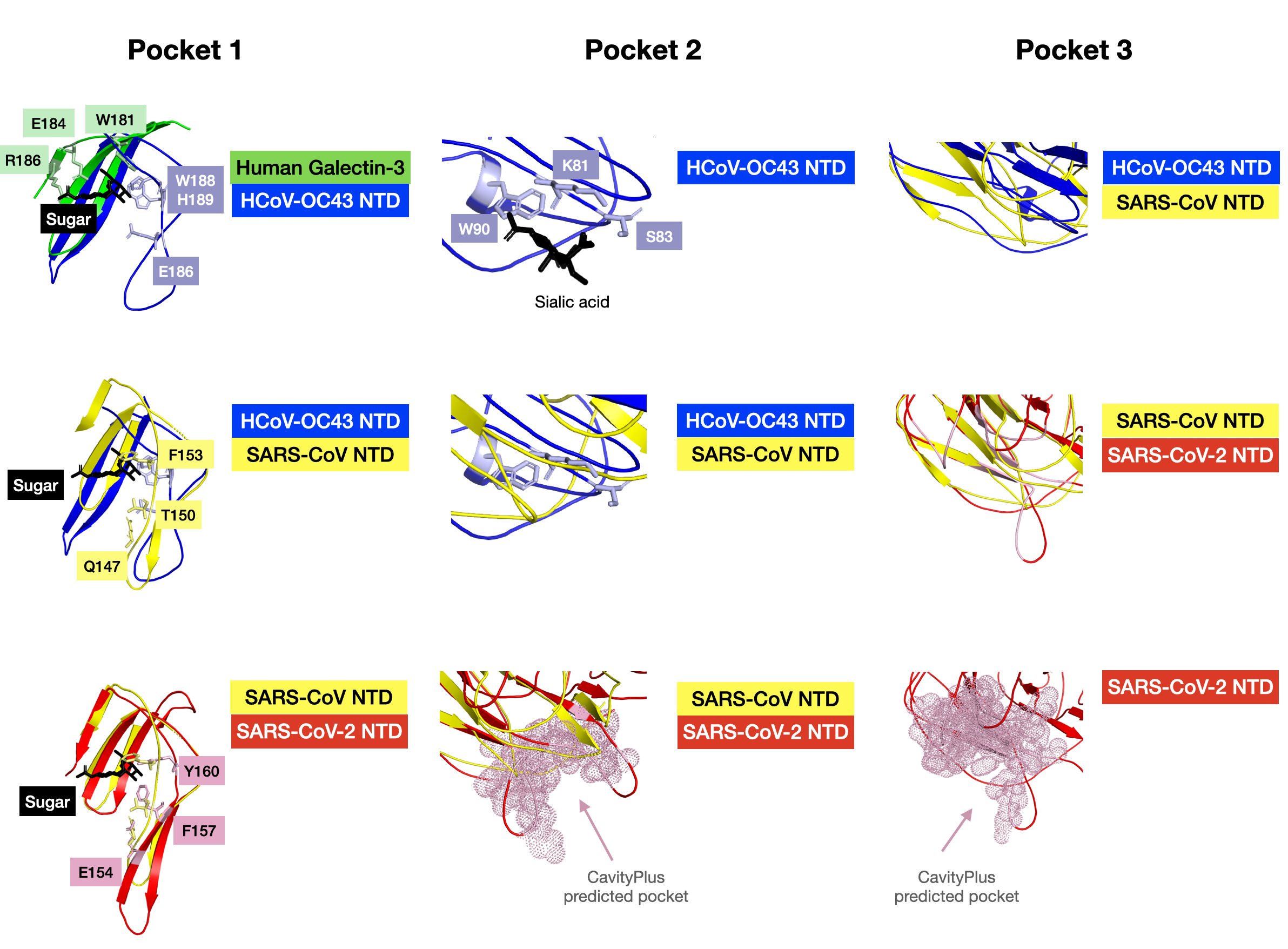


**Supplementary Figure 2.** Evolution of the sugar-binding pocket from human Galectin-3 to SARS-CoV-2 and emergence of other sugar-binding regions. We only show the sugar-binding regions. We coloured the human Galectin-3 NTD structure in green, hCoV-OC42 NTD structure in blue, SARS-CoV NTD structure in yellow and SARS-CoV-2 NTD structure in red. PDB structures 1A3K,7C2L,6ACC, 6NZK respectively. We also compared against the human Galectin-3 protein which is in the same fold group as this superfamily. It can be seen that loop insertions in the BCoVs near the sugar-binding pocket 1 appear to enhance the contact of the protein with the ligand and the contacts increase going from human Galectin-3 to HCoV-OC43 (a BCoV that emerged in the 19th century) up to SARS-CoV-2. We also see loop extensions when comparing SARS-CoV-2 to human Galectin-3 in the region around pocket 2 (see Supplementary Table 5). Human Galectin-3 has been shown not to bind sialic acids at this pocket . These loops seem to be highly structurally variable and this pocket is less well defined in SARS-CoV. Comparing the structures and sequences of HCoV-OC43 and SARS-CoV-2 we see strong innovation in pocket 2 in SARS-CoV-2 suggesting alterations in this pocket were important for the evolution of this virus. Similarly, structural analyses comparing pocket 3 across the coronaviruses suggests that this pocket can be highly variable too, and may offer another potential sialic acid binding site.


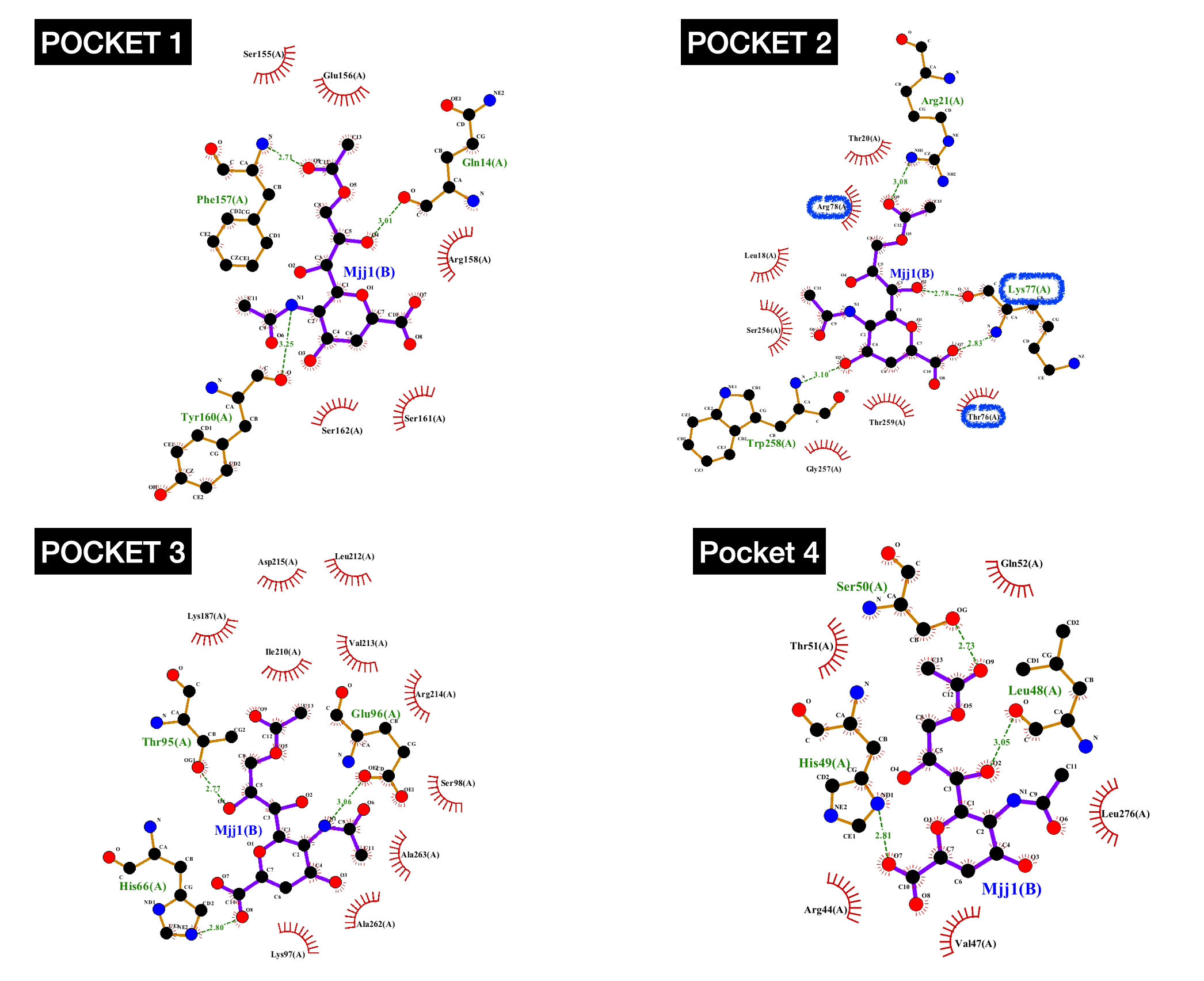


**Supplementary Figure 3.** LigPlot showing H-bonding interactions of sialic acid with pockets 1, 2, 3 and 4 of SARS-CoV2 NTD. Residues highlighted in blue involve the sugar binding motif (72-GTNGTKR-78). For pockets 1 and 4, we observe fewer SARS-CoV-2 NTD residues forming hydrogen bonds and Van der Waals interactions with sialic acid compared to pocket 2 and 3, in agreement with the lower predicted binding energy for these pockets.


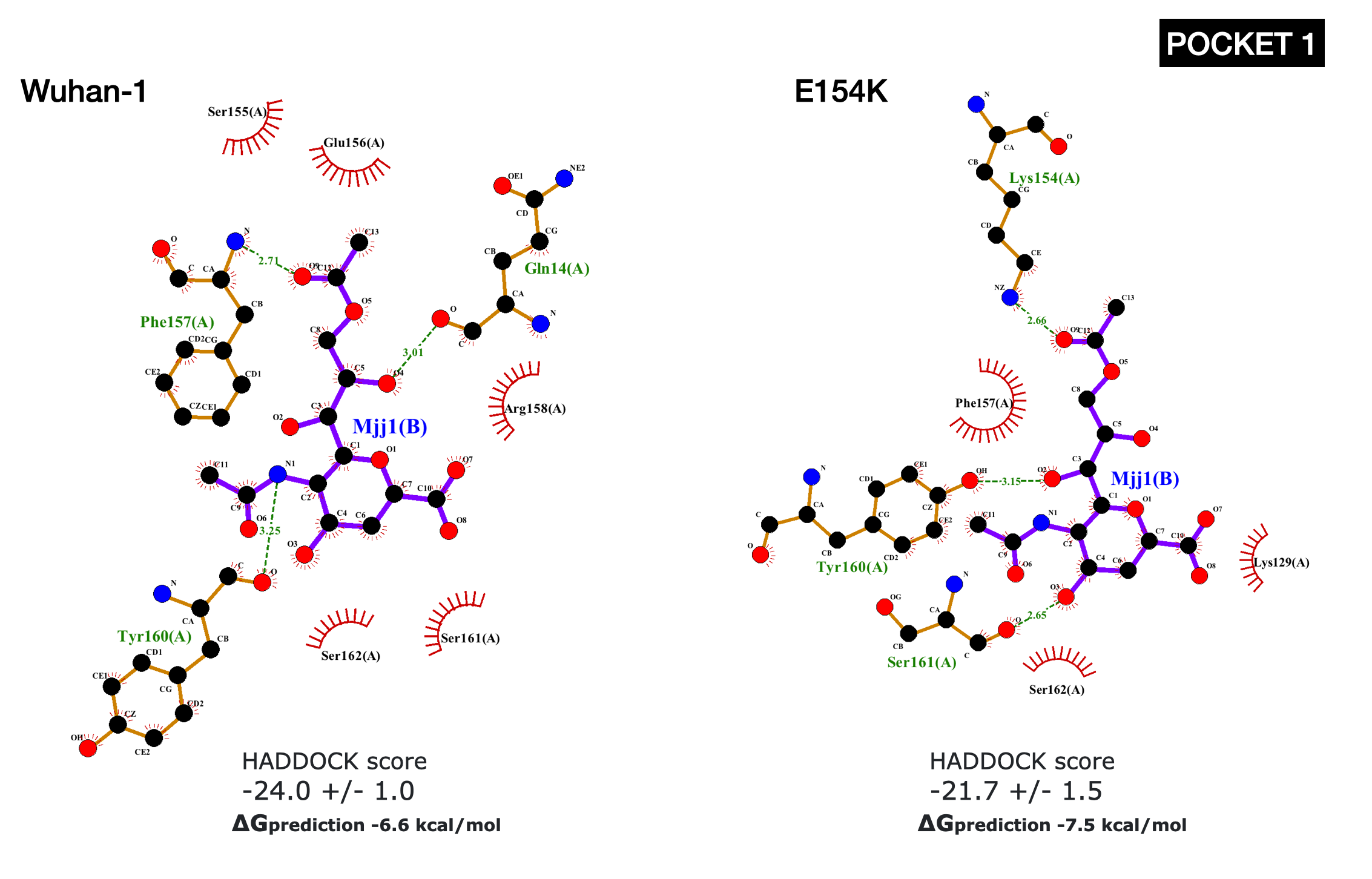


**Supplementary Figure 4.** LigPlots of SARS-COV-2 Wuhan-1 NTD and SARS-COV-2 E154 NTD mutant with sialic acid (Pocket 1).


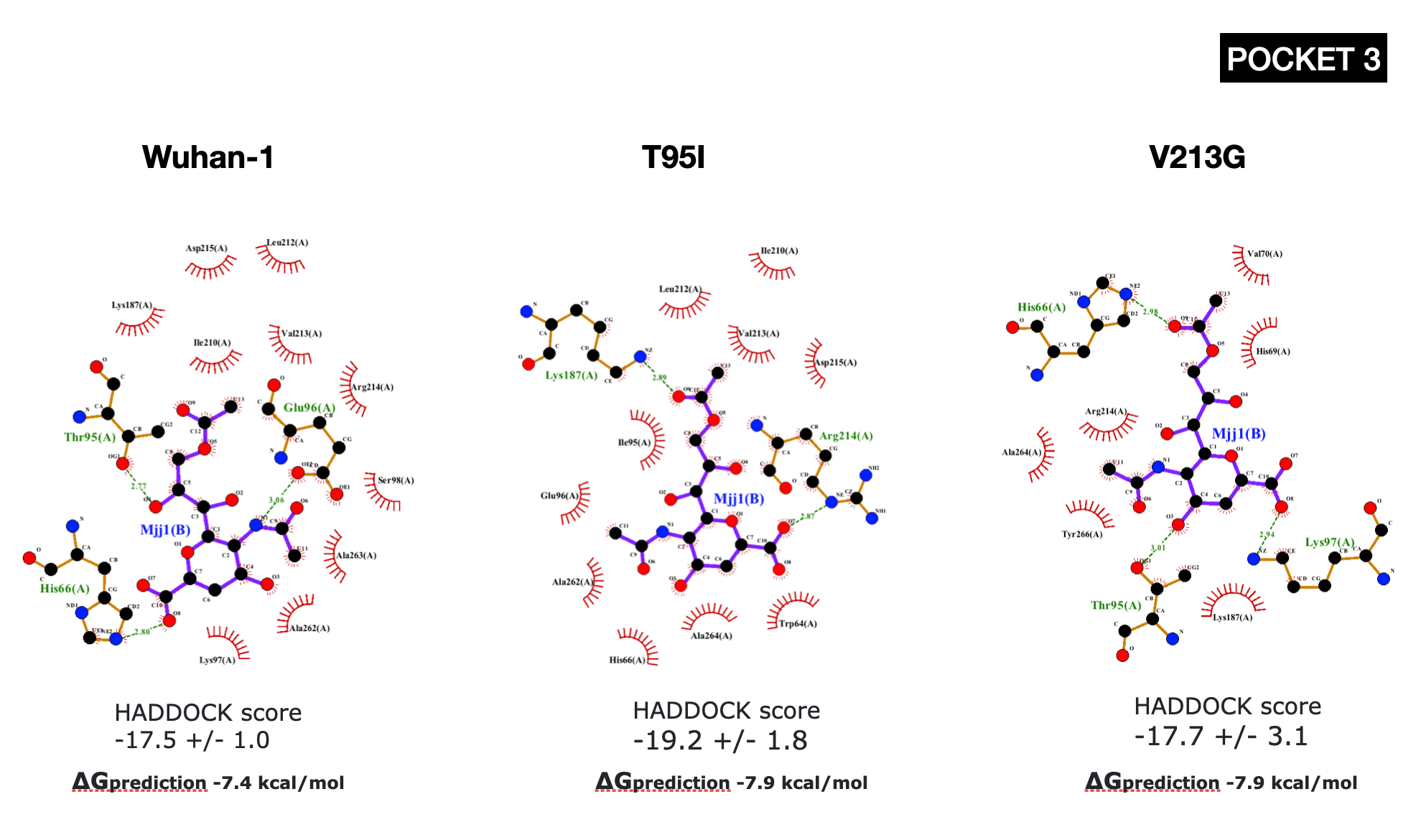


**Supplementary Figure 5.** LigPlots of SARS-COV-2 Wuhan-1 NTD, SARS-COV-2 Wuhan-1 T95I NTD mutant and SARS-CoV-2 V213G mutant with sialic acid (Pocket 3).


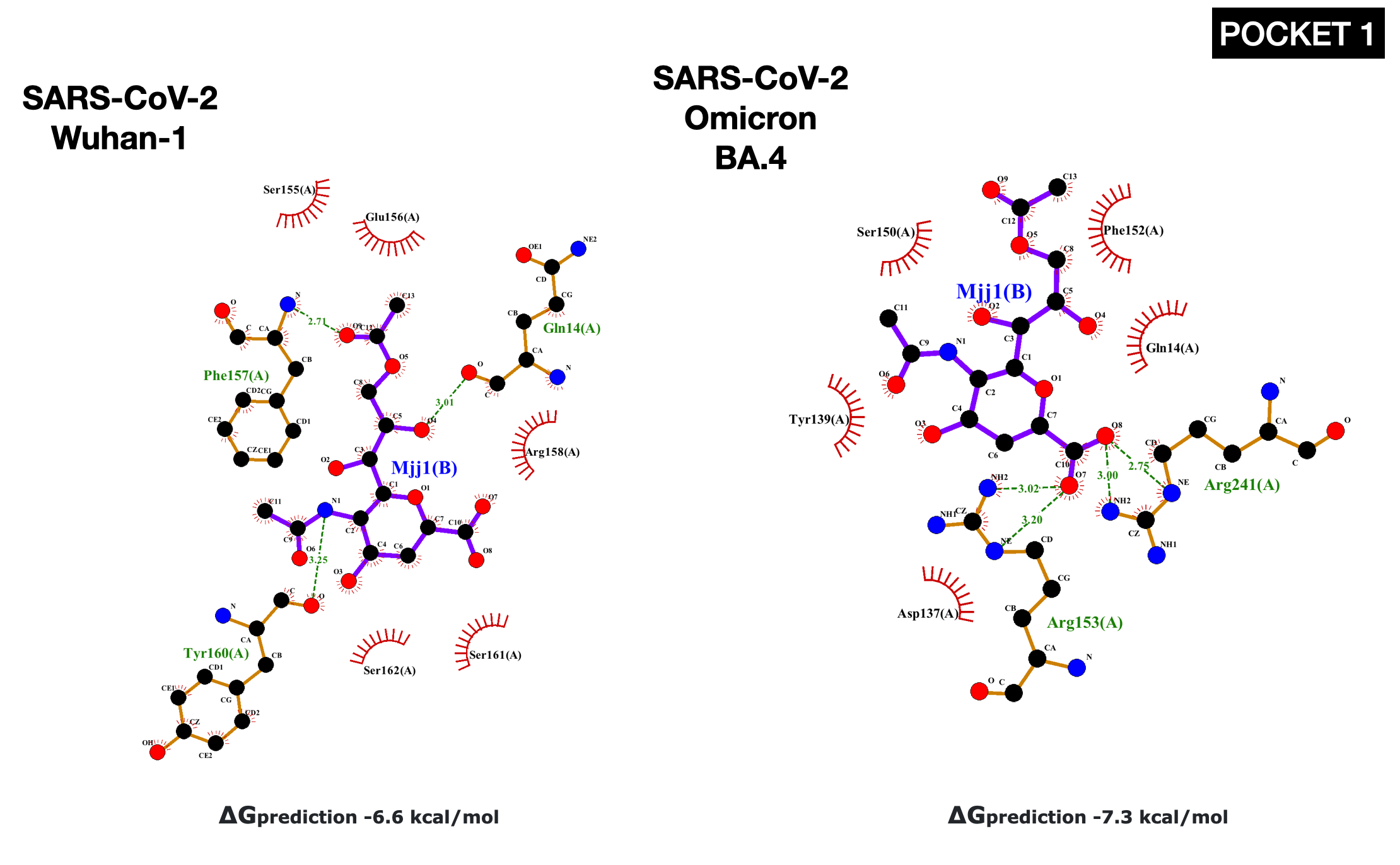


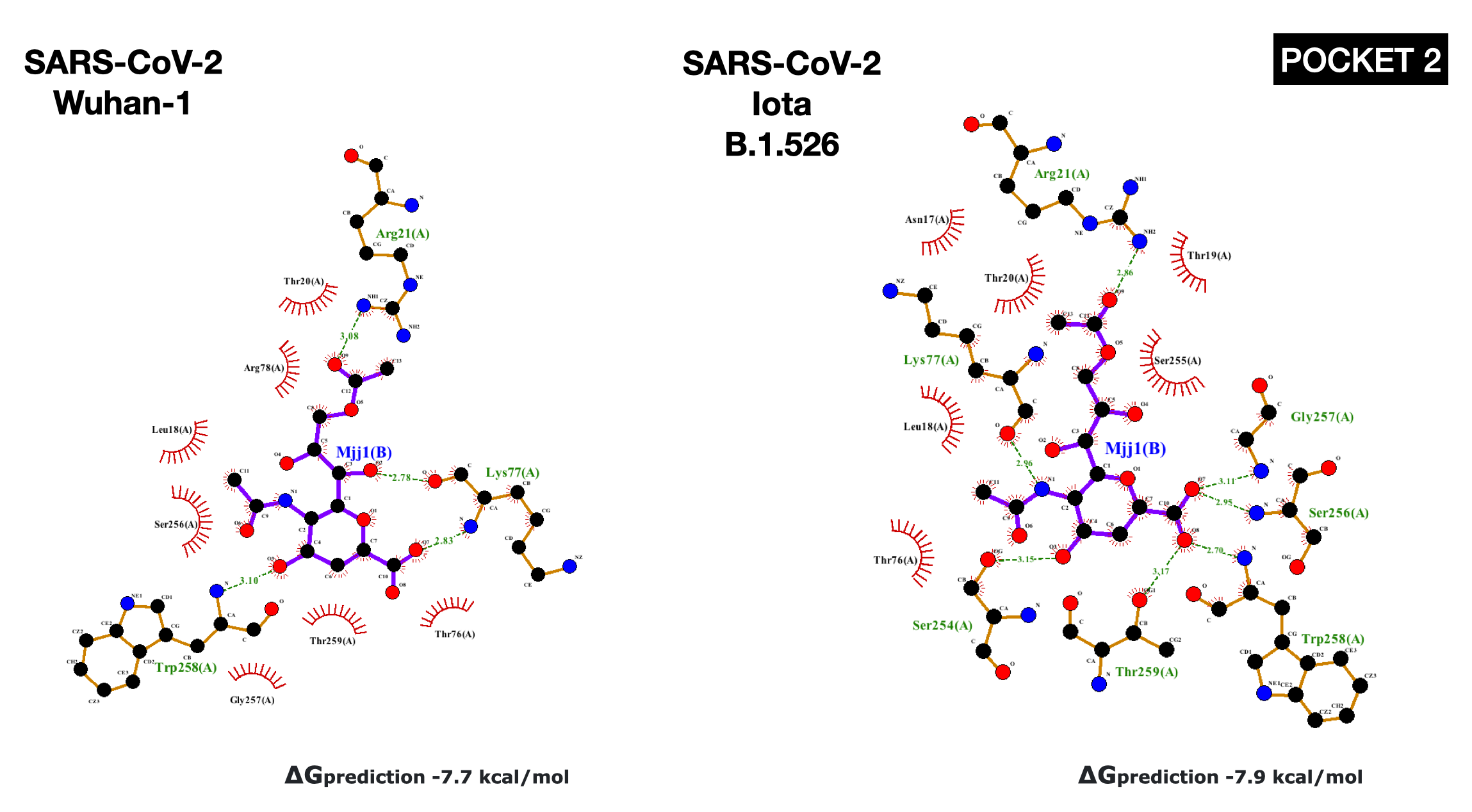


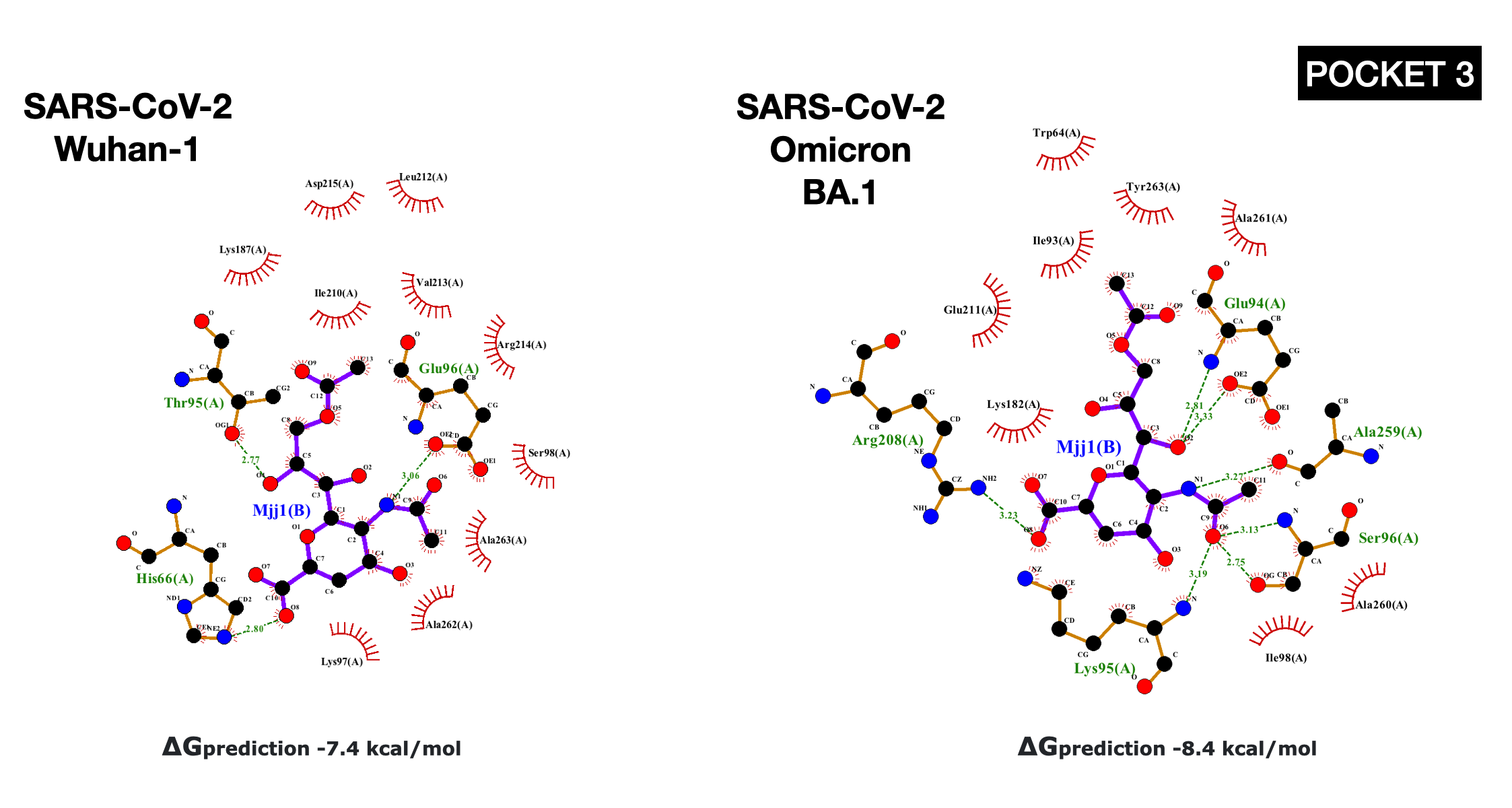


**Supplementary Figure 6**. LigPlots of SARS-CoV-2 Wuhan-1 and the strain that shows highest deviation for each pocket (Pocket 1: SARS-CoV-2 wild Wuhan versus SARS-CoV-2, BA.4, Omicron variant; Pocket 2: SARS-CoV-2 wild Wuhan versus SARS-CoV-2, B.1.526, Iota variant; Pocket 3: SARS-CoV-2 wild Wuhan versus SARS-CoV-2, B.1.1.263 and BA.1, Omicron variant). For pocket 1, there is an increase from 3 hydrogen bonds in the Wuhan-1 strain to 4 hydrogen bonds in the Omicron variant. For pocket 2, there is an increase from 4 hydrogen bonds in the Wuhan-1 strain to 7 hydrogen bonds in the Iota variant. For pocket 3, there is an increase from 3 hydrogen bonds in the Wuhan-1 strain to 7 hydrogen bonds in the Omicron variant.


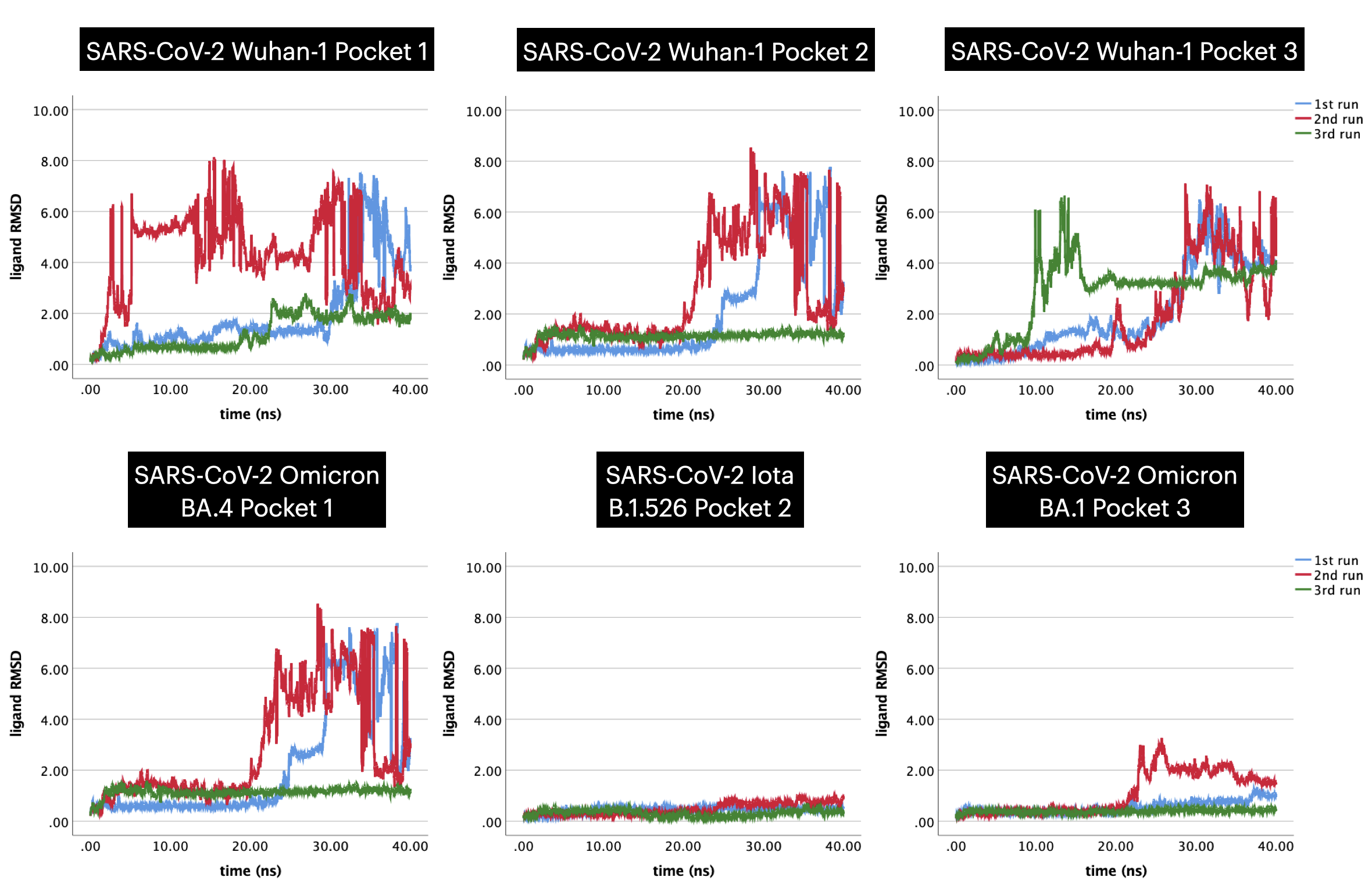


**Supplementary Figure 7**. Ligand RMSD of the original Wuhan-1 strain and the strain that shows highest deviation (Pocket 1: BA.4, Omicron variant; Pocket 2: B.1.526, Iota variant; Pocket 3: SARS-CoV-2, B.1.1.263 and BA.1, Omicron variant) to sialic acid for all 3 pockets.


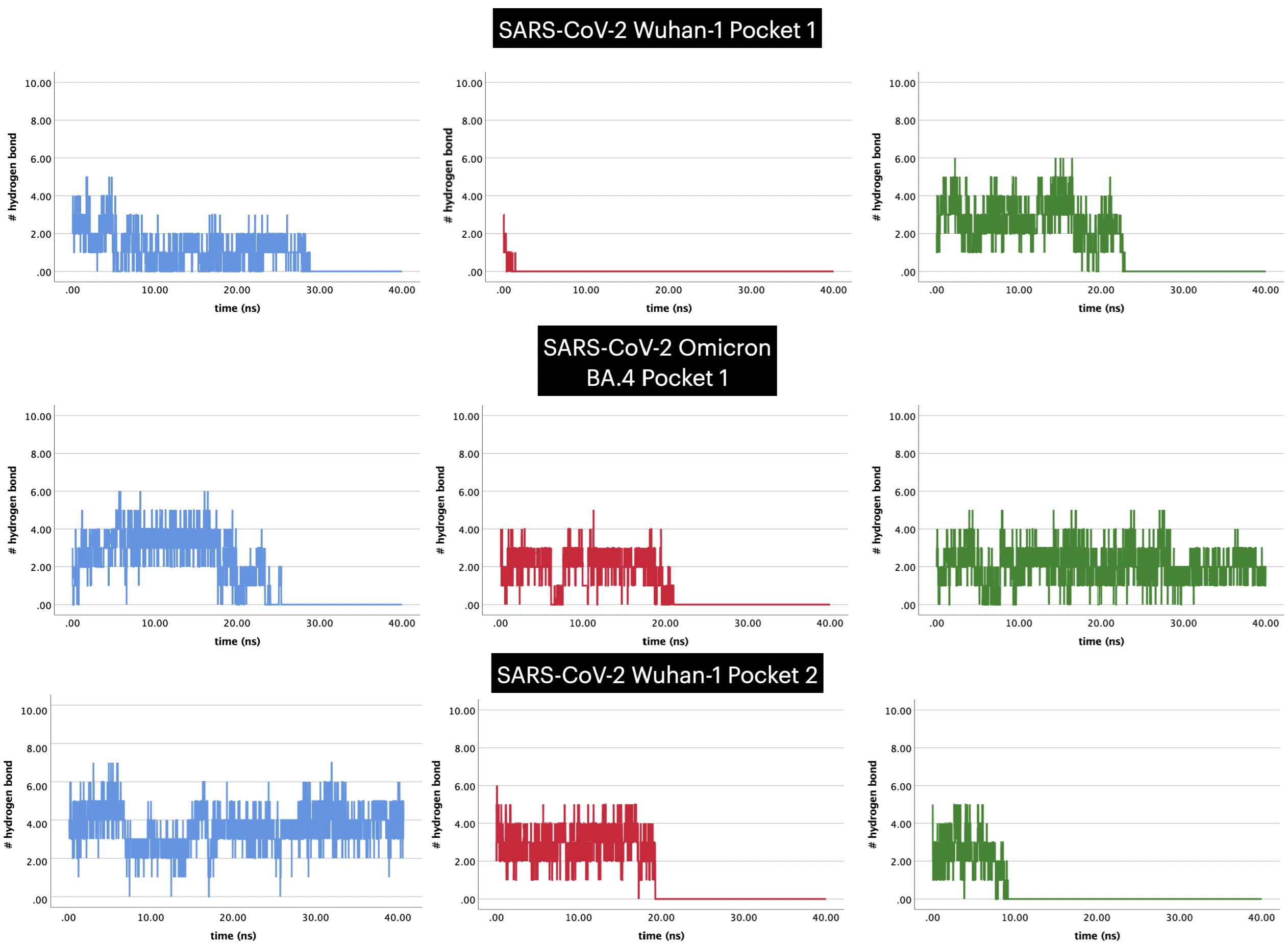


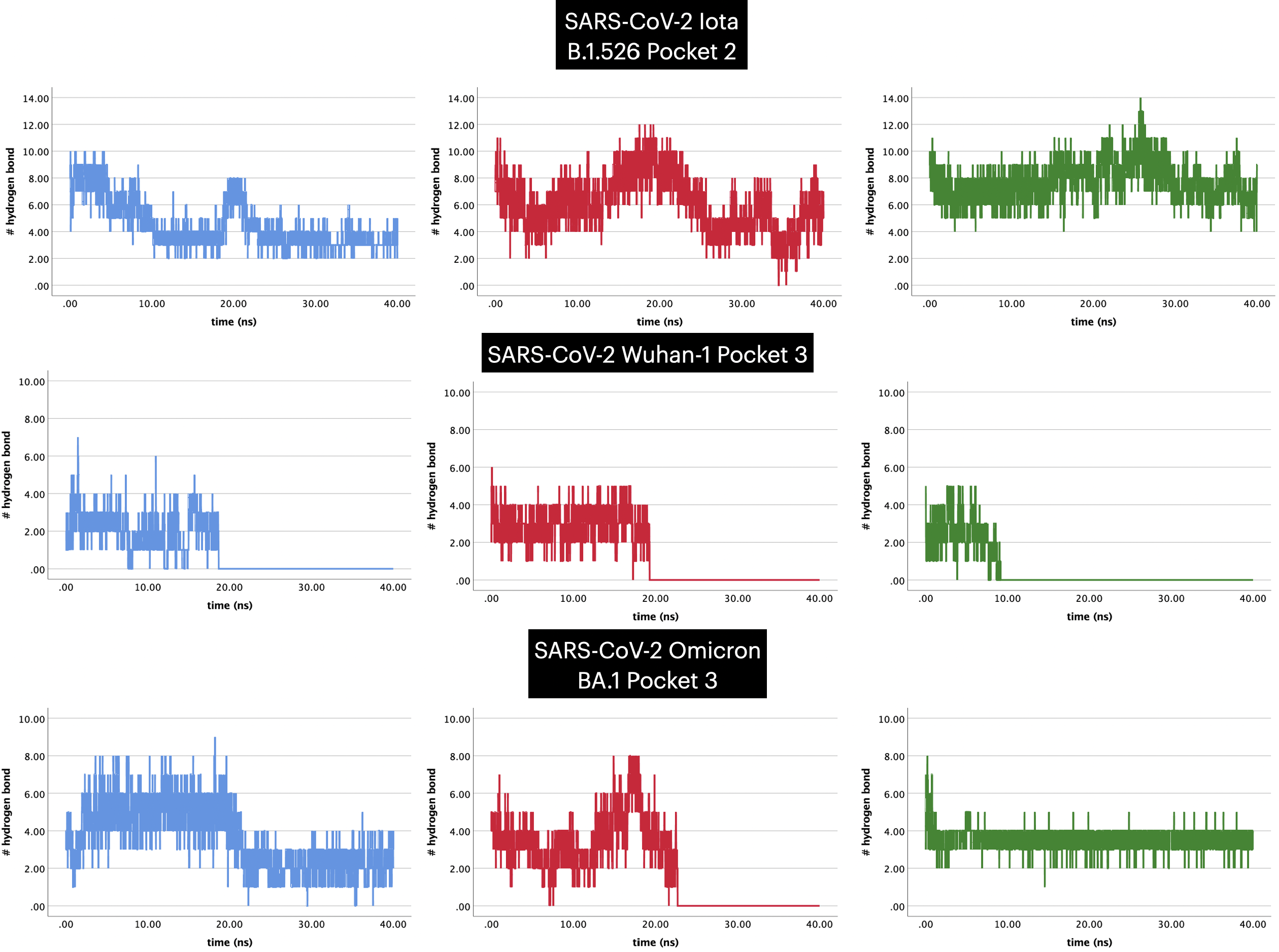


**Supplementary Figure 8**. Time evolution of the Number of hydrogen bond formed between the original Wuhan-1 strain and the strain that shows highest deviation (Pocket 1: BA.4, Omicron variant; Pocket 2: B.1.526, Iota variant; Pocket 3: SARS-CoV-2, B.1.1.263 and BA.1, Omicron variant) to sialic acid for all 3 pockets. The first run is coloured blue, the second run is coloured red and the third is coloured green.


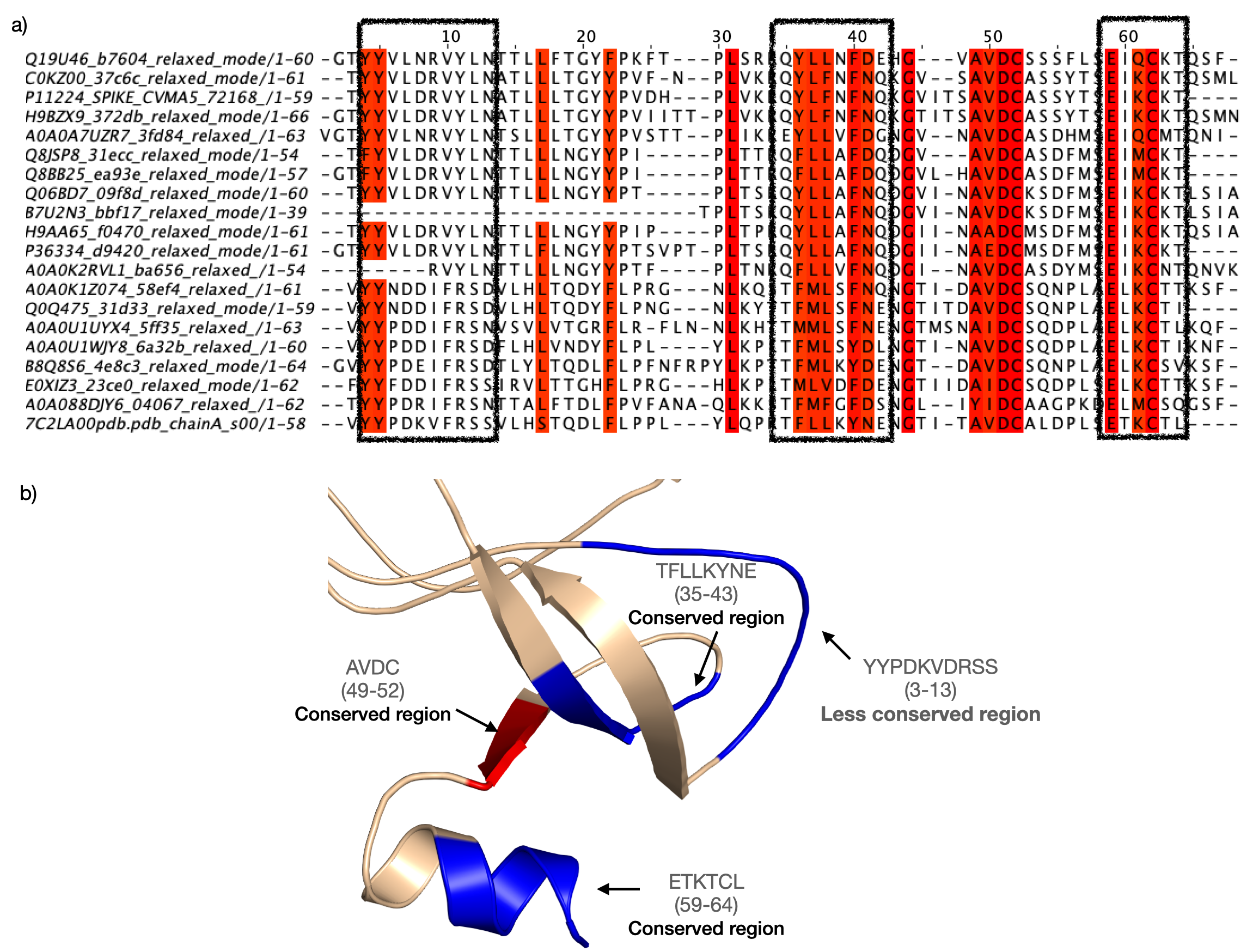


**Supplementary Figure 9.** Sequence conservation of NTD pocket 4 residues. A) A structure-based multiple sequence alignment using structures covering pocket 4 and residues that are in the vicinity of it. We coloured all the alignment positions with ScoreCons value above 70 as red. We also highlighted the CavityPlus druggable residues in the box. B) We coloured the druggable residues of the SARS-CoV-2 NTD structure in blue and a highly conserved region in red.

**Supplementary Table 1.** NCBI/GISAID ID and common name of coronavirus sequences used in this study.

| **NCBI/GISAID ID** | **Name** |
| --- | --- |
| EPI_ISL_412977 | Bat coronavirus RmYN02 (BatCoV-RmYN02) |
| AVP78042.1 | Bat coronavirus ZXC21 (BatCoV-ZXC21) |
| MG772933.1 | Bat coronavirus ZC45 (BatCoV-ZC45) |
| QHR63300.2 | Bat coronavirus RaTG13(BatCoV-RaTG13) |
| AAU04646.1 | Civet severe acute respiratory syndrome (SARS)-associated coronavirus (Civet-SARSr-Cov) |
| AAP13441.1 | SARS-associated coronavirus (SARS-CoV) |
| QIA48623.1 | Pangolin coronavirus GX (PangolinCoV-GX) |
| QIQ54048.1 | Vero E6 cell passaged Pangolin coronavirus GX (PangolinCoV-GX Vero) |
| QIG55945.1 | Pangolin coronavirus GD (PangolinCoV-GD) |
| AAR01015.1 | Human coronavirus OC43 (HCoV-OC43) |
| KJ156866.1 | Middle East respiratory syndrome–related coronavirus (MERS-CoV) |
| YP_009724390.1 | SARS-CoV-2 (Wuhan-Hu-1 strain) |
| EPI_ISL_741243 | SARS-CoV-2 B.1.1.7 (Alpha variant) |
| EPI_ISL_736967 | SARS-CoV-2 B.1.351 (Beta variant) |
| EPI_ISL_804832 | SARS-CoV-2 P.1 (Gamma variant) |
| EPI_ISL_1068256 | SARS-CoV-2 P.1 (Gamma variant) |
| EPI_ISL_1219135 | SARS-CoV-2 P.1 (Gamma variant) |
| EPI_ISL_2521822 | SARS-CoV-2 B.1.617.1 (Kappa variant) |
| EPI_ISL_2521781 | SARS-CoV-2 B.1.617.2 (Delta variant) |
| EPI_ISL_6656070 | SARS-CoV-2 AY.1 (Delta Plus variant) |
| EPI_ISL_5811040 | SARS-CoV-2 AY.2 (Delta Plus variant) |
| EPI_ISL_6695006 | SARS-CoV-2 AY.3 (Delta Plus variant) |
| EPI_ISL_1970532 | SARS-CoV-2 B.1.617.3 |
| EPI_ISL_2801732 | SARS-CoV-2 B.1.429+B.1.427 (Epsilon variant) |
| EPI_ISL_3692979 | SARS-CoV-2 B.1.526 (Iota variant) |
| EPI_ISL_3574438 | SARS-CoV-2 B.1.525 (Eta variant) |
| EPI_ISL_3147606 | SARS-CoV-2 B.1.620 |
| EPI_ISL_3654107 | SARS-CoV-2 B.1.621 (Mu variant) |
| EPI_ISL_2535683 | SARS-CoV-2 P.3 (Theta variant) |
| EPI_ISL_3672611 | SARS-CoV-2 C.37 (Lambda variant) |
| EPI_ISL_1181371 | SARS-CoV-2 N.10 |
| EPI_ISL_6640916 | SARS-CoV-2 B.1.1.263 |
| EPI_ISL_6704875 | SASR-CoV-2 BA.1 (Omicron variant) |
| EPI_ISL_9087709 | SASR-CoV-2 BA.2 (Omicron variant) |
| EPI_ISL_12497122 | SASR-CoV-2 BA.4 (Omicron variant) |
| EPI_ISL_12112497 | SASR-CoV-2 BA.5 (Omicron variant) |

**Supplementary Table 2.** Structure modelling template, structural template sequence identity and model quality (normalised DOPE) of BCoV Spike protein NTD models using FunMod modelling platform

| **NTD model** | **Template** | **Sequence Identity** | **Normalised DOPE** |
| --- | --- | --- | --- |
| BatCoV-RmYN02 | 7CN8 | 47% | -0.36 |
| BatCoV-ZXC21 | 7CN8 | 65% | -0.86 |
| BatCoV-ZC45 | 7CN8 | 63% | -0.80 |
| Civet-SARSr-Cov | 6ACC | 100% | -0.60 |
| Pangolin-CoV-Gx-Vero | 7CN8 | 97% | -0.58 |
| SARS-CoV-2 B.1.1.7 | 7C2L | 98 % | -0.39 |
| SARS-CoV-2 B.1.351 | 7C2L | 95 % | -0.44 |
| SARS-CoV-2 P.1 (EPI_ISL_804832) | 7C2L | 99 % | -0.45 |
| SARS-CoV-2 P.1 (EPI_ISL_1068256) | 7C2L | 98 % | -0.37 |
| SARS-CoV-2 P.1 (EPI_ISL_1219135) | 7C2L | 97 % | -0.31 |
| SARS-CoV-2 B.1.617.1 | 7C2L | 99 % | -0.37 |
| SARS-CoV-2 B.1.617.2 | 7C2L | 99 % | -0.37 |
| SARS-CoV-2 AY.1 | 7C2L | 96 % | -0.31 |
| SARS-CoV-2 AY.2 | 7C2L | 97% | -0.31 |
| SARS-CoV-2 AY.3 | 7C2L | 97 % | -0.32 |
| SARS-CoV-2 B.1.617.3 | 7C2L | 99 % | -0.43 |
| SARS-CoV-2 B.1.429+B.1.427 | 7C2L | 99 % | -0.39 |
| SARS-CoV-2 B.1.526 | 7C2L | 99 % | -0.34 |
| SARS-CoV-2 B.1.525 | 7C2L | 97 % | -0.47 |
| SARS-CoV-2 B.1.620 | 7C2L | 94 % | -0.51 |
| SARS-CoV-2 B.1.621 | 7C2L | 83 % | -0.19 |
| SARS-CoV-2 P.3 | 7C2L | 98 % | -0.33 |
| SARS-CoV-2 C.37 | 7C2L | 99 % | -0.44 |
| SARS-CoV-2 N.10 | 7C2L | 96% | -0.46 |
| SARS-CoV-2 B.1.1.263 and BA.1 (Omicron) | 7C2L | 93% | -0.48 |
| SARS-CoV-2 BA.2 (Omicron) | 7C2L | 98% | -0.42 |
| SARS-CoV-2 BA.4 (Omicron) | 7C2L | 95% | -0.45 |
| SARS-CoV-2 BA.5 (Omicron) | 7C2L | 96% | -0.49 |

**Supplementary Table 3.** Predicted lDDT scores of AlphaFold2 BCoV Spike protein NTD models. Models with a predicted lDDT score above 70 are considered to be acceptable.

| **UniProt ID** | **lDDT score** |
| --- | --- |
| A0A088DJY6 | 79.65 |
| A0A0A7UZR7 | 95.36 |
| A0A0K1Z074 | 94.93 |
| A0A0K2RVL1 | 93.97 |
| A0A0U1UYX4 | 94.32 |
| A0A0U1WJY8 | 89.79 |
| B7U2N3 | 93.06 |
| B8Q8S6 | 85.63 |
| C0KZ00 | 89.85 |
| E0XIZ3 | 94.14 |
| H9AA65 | 94.41 |
| H9BZX9 | 88.48 |
| P11224 | 95.65 |
| P36334 | 95.64 |
| Q06BD7 | 94.19 |
| Q0Q475 | 93.45 |
| Q19U46 | 89.49 |
| Q77NQ7 | 91.86 |
| Q8BB25 | 96.78 |
| Q8JSP8 | 96.58 |
| R9QTA0 | 88.90 |

**Supplementary Table 4**. Residues in the known and putative sugar binding pockets of SARS-CoV-2

| **Function** | **Note** | **Viruses that contain the pocket** | **Pocket** | **Pocket Residues** | **Citations** |
| --- | --- | --- | --- | --- | --- |
| Sialic acid/ ganglioside binding | Sialic acid-binding region (*In silico* structural and molecular modelling study) | Identified by analysis of SARS-CoV-2 | 1 | D111, S112, K113, Q134, F135, C136, N137, F140, G142, E156, F157, R158, Y160, S161, S162 | Fantini et al., 2020 [1] |
| Sugar binding | BcoV sugar-binding domain (identified by analysis of bovine coronavirus) | BovineCoV-NTD, PHEV-NTD, HCoV-OC43-NTD, HCoV-HKU23-NTD, HKU1-NTD, MHV-NTD | 1 | E154, F157, Y160 | Cheng et al., 2019; Behloul et al., 2020 [2,3] |
| Sugar binding | Sugar receptor-interacting motif (identified by analysis of bacteriophage CBA120, infectious bronchitis coronavirus) | BovineCoV-NTD, PHEV-NTD, HCoV-OC43-NTD, HCoV-HKU23-NTD, HKU1-NTD, MHV-NTD | 2, 3 | G72, T73, N74, G75, T76, K77, R78 | Cheng et al., 2019; Behloul et al., 2020 [2,3] |
| Sialic acid-binding | Identified by analysis of SARS-CoV-2 – similar to those reported for HCoV-OC43 structure with sialic-acid bound | BovineCoV-NTD, PHEV-NTD, HCoV-OC43-NTD, HCoV-HKU23-NTD, HKU1-NTD, MHV-NTD | 2 | R21, Q23, L24, H69, F79, P82, R246 | Cheng et al., 2019; Tortorici et al., 2019; Baker et al., 2021 [2,4,5] |
| Sialic acid-binding | Universal saturation transfer analysis (NMR method) and identified density regions corresponding to sialoside binding sites using HADDOCK. | Identified by analysis of SARS-CoV-2 | 2 | L18, H69, F79, Y145, W152, Q183, L249, T259 | Buchanan et al., 2022 [6] |
| Druggable Pocket | Predicted using SiteMap (PDB ID 7JJI) | Identified by analysis of SARS-CoV-2 | 2 | R21, T22, Q23, L24, P26, R78, P82, V83, L110, F135, C136, N137, R237 | Di Gaetano et al., 2021[7] |
| Druggable pocket | Predicted using CavityPlus (PDB ID 7C2L) | Identified by analysis of SARS-CoV-2 | 2 | V16, N17, L18, T19, T20, R21, T22, I68, H69, N74, G75, T76, K77, R78, F79, D80, L244, S247, S256, G257, W258, T259, A260 | This study |
| Druggable Pocket | Predicted using SiteMap (PDB ID 7JJI) | Identified by analysis of SARS-CoV-2 | 3 | F92, S94, E96, K97, S98, R102, N121, V126, I128, M177, D178, K182, N188, R190, F192, I203, L226, V227, L229 | (Di Gaetano et al., 2021)[7] |
| Druggable pocket | Predicted using CavityPlus (PDB ID 7C2L) | Identified by analysis of SARS-CoV-2 | 3 | A27, W64, F65, H66, A67, I68, H69, V70, S71, G72, A93, S94, T95, E96, K97, S98, N99, I100, N185, F186, K187, N188, L189, I210, N211, L212, V213, D214, D215, L216, P217, A260, G261, A262, A263, A264, Y265, Y266 | This study |
| Druggable pocket | Predicted using CavityPlus (PDB ID 7C2L) | Identified by analysis of SARS-CoV-2 | 4 | Y38, P39, D40, K41, V42, F43, R44, S45, S46, V47, L48, H49, S50, T51, Q52, D53, T274, F275, L276, L277, K278, Y279, N280, E281, C291, E298, T299, K300, C301, T302, L303 | This study |

**Supplementary Table 5.** PRODIGY predicted binding energy of sialic acid to NTD pockets 2 and 3 of selected BCoVs. None of these proteins binds sialic acid as strongly as SARS-CoV-2 (see Table 2). Previous structural analysis by Cheng et al. also found pocket 2 of SARS-CoV to be less-defined than other BCoVs [2]. We also compared the LigPlots for sialic acid docked to SARS-CoV and Pangolin-CoV-GX with those for SARS-CoV-2 (See Supplementary Figure 3). For both pockets 2 and 3, we observe more interactions with sialic acid and more hydrogen bonds formed in SARS-CoV-2 NTD than SARS-CoV NTD. The number of sialic acid interacting residues for Pangolin-CoV-GX and SARS-CoV-2 are similar.

| Virus | PRODIGY predicted binding energy (kcal/mol) | |
| --- | --- | --- |
|  | Pocket 2 | Pocket 3 |
| SARS-CoV | -6.4 | -6.6 |
| Pangolin-CoV-GX | -7.3 | -7.2 |
| SARS-CoV-2 | -7.7 | -7.4 |

**Supplementary Table 6.** In silico mutagenesis performed on the sialic acid binding residues of pocket 2. Mutant with an increased binding affinity of ≥0.5 kcal/mol is coloured red. We found weaker binding for all the mutants investigated for pocket 2, with significant change of ≥ 0.5 kcal/mol for mutants R78A and T259A.

| Structure | Predicted binding energy | Difference |
| --- | --- | --- |
| Wuhan-1 | -7.7 kcal/mol | NA |
| L18A | -7.6 kcal/mol | -0.1 |
| T20A | -7.5 kcal/mol | -0.2 |
| R21A | -7.5 kcal/mol | -0.2 |
| T76A | -7.4 kcal/mol | -0.3 |
| K77A | -7.3 kcal/mol | -0.4 |
| R78A | -7.1 kcal/mol | -0.6 |
| S256A | -7.4 kcal/mol | -0.1 |
| G257A | -7.4 kcal/mol | -0.1 |
| W258A | -7.3 kcal/mol | -0.4 |
| T259A | -7.2 kcal/mol | -0.5 |

**Supplementary Table 7.** In silico mutagenesis performed on the sialic acid binding residues of Pocket 3. There are no significant impacts for mutations of binding residues in Pocket 3.

| Structure | Predicted binding energy | Difference |
| --- | --- | --- |
| Wuhan-1 | -7.4 kcal/mol | NA |
| H66A | -7.3 kcal/mol | -0.1 |
| T95A | -7.4 kcal/mol | 0 |
| E96A | -7.3 kcal/mol | -0.1 |
| K97A | -7.4 kcal/mol | 0 |
| S98A | -7.5 kcal/mol | 0.1 |
| K187A | -7.3 kcal/mol | -0.1 |
| I210A | -7.6 kcal/mol | 0.2 |
| L212A | -7.4 kcal/mol | 0 |
| V213A | -7.6 kcal/mol | 0.2 |
| R214A | -7.3 kcal/mol | -0.1 |
| D215A | -7.5 kcal/mol | 0.1 |

**Supplementary Table 8.** NTD mutations found in SARS-CoV-2 Variants of Concern (VOC)/Interest (VOI) and present in NTD sugar binding pockets 1 to 3.

| Name | Variant Classification | NTD mutation/deletion | NTD mutation lies close to sugar-binding pocket |
| --- | --- | --- | --- |
| Alpha variant (B.1.1.7) | VOC | H69del, V70del, Y144del | Pocket 1: Y144del  Pocket 2: H69del, V70del  Pocket 3: H69del, V70del |
| Beta variant (B.1.351) | VOC | L18F, D80A, D215G, L241del, A242del, L243del | Pocket 1: L18F  Pocket 2: L18F, D80A, L241del, L242del, A243del,  Pocket 3: D215G |
| Gamma variant (P.1)  EPI_ISL_804832 | VOC | L18F, T20N, P26S, D138Y, R190S | Pocket 1: L18F, D138Y  Pocket 2: L18F, T20N, D80A  Pocket 3: P26S, R190S |
| Gamma variant (P.1)  EPI_ISL_1219135 | VOC | L18F, T20N, P26S, D138Y, N188S, L189del, R190del | Pocket 1: L18F, D138Y  Pocket 2: L18F, T20N,  Pocket 3: P26S, N188S, L189del, R190del |
| Gamma variant (P.1)  EPI_ISL_1068256 | VOC | L18F, P26S, D138Y, ins214ANRN | Pocket 1: L18F, D138Y  Pocket 2: L18F  Pocket 3: P26S, ins214ANRN |
| Kappa variant  (B.1.617.1) | VOI | T95I, E154K | Pocket 1: E154K  Pocket 3: T95I |
| Delta variant (B.1.617.2) | VOC | T19R, T95I | Pocket 1: T19R  Pocket 2: T19R  Pocket 3: T95I |
| Delta Plus variant  (AY.1) | VOC | T19R, H49Y, T95I, G142D, E156G, F157del, R158del, W258L | Pocket 1: T19R, G142D, E156G, F157del, R158del  Pocket 2: T19R, W258L  Pocket 3: T95I |
| Delta Plus variant  (AY.2) | VOC | T19R, T95I, G142D, E156G, F157del, R158del, W258L | Pocket 1: T19R, E156G, F157del, R158del  Pocket 2: T19R, W258L  Pocket 3: T95I |
| Delta Plus variant  (AY.3) | VOC | T19R, E156G, F157del, R158del | Pocket 1: T19R, E156G, F157del, R158del  Pocket 2: T19R |
| B.1.617.3 | VOI | T19R, G142D | Pocket 1: T19R, G142D  Pocket 2: T19R |
| Epsilon variant (B.1.429+B1.427) | VOI | S13I (signal peptide), W152C | Pocket 1: W152C |
| Eta variant (B.1.525) | VOI | Q52R, A67V, H69del, V70del, Y144del | Pocket 1: Y144del  Pocket 2: A67V, H69del, V70del  Pocket 3: A67V, H69del, V70del |
| Iota variant (B.1.526) | VOI | L5F (signal peptide), T95I, D253G | Pocket 2: D253G  Pocket 3: T95I |
| B.1.620 | VOI | P26S, H69del, V70del, V126A, Y144del, L242del, A243del, L244del, H245Y | Pocket 1: Y144del  Pocket 2: H69del, V70del, L242del, A243del, L244del, H245Y  Pocket 3: H69del, V70del, P26S |
| B.1.621 | VOI | T95I, Y144S, Y145N | Pocket 1: Y144S, Y145N  Pocket 3: T95I |
| Lambda variant (C.37) | VOI | G75V, T76I, R246del, S247del, Y248del, L249del, T250del, P251del, G252del, D253N | Pocket 2: G75V, T76I, R246del, S247del, Y248del, L249del, T250del, P251del, G252del, D253N |
| Theta variant (P.3) | VOI | L141del, G142del, V143del | Pocket 1: L141del, G142del, V143del |
| N.10 |  | P9L (signal peptide), L141del, G142del, V143del, Y144del, I210V, L212I, N211del, S256del, G257del, W258del | Pocket 1: L141del, G142del, V143del, Y144del  Pocket 2: S256del, G257del, W258del  Pocket 3: I210V, L212I, N211del, |
| B.1.1.263 and  Omicron variant (BA.1)  (Both strains have identical NTD mutations) | VOC | A67V, H69del, V70del, T95I, G142D, V143del, Y144del, Y145del, N211del, L212I, ins214EPE | Pocket 1: G142D, V143del, Y144del, Y145del  Pocket 2: H69del, V70del,  Pocket 3: A67V, H69del, V70del, T95I, N211del, L212I, ins214EPE |
| Omicron variant (BA.2) | VOC | T19I, L24del, P25del, P26del, A27S, G142D, V213G | Pocket 1: T19I, G142D  Pocket 2: T19I, L24del, P25del, P26del, A27S  Pocket 3: V213G |
| Omicron variant (BA.4) | VOC | V3G (signal peptide), T19I, L24del, P25del, P26del, A27S, H69del, V70del, G142D, V213G | Pocket 1: T19I, G142D  Pocket 2: T19I, L24del, P25del, P26del, A27S, H69del, V70del  Pocket 3: H69del, V70del, V213G |
| Omicron variant (BA.5) | VOC | T19I, L24del, P25del, P26del, A27S, H69del, V70del, T76I, G142D, V213G | Pocket 1: T19I, G142D  Pocket 2: T19I, L24del, P25del, P26del, A27S, H69del, V70del  Pocket 3: H69del, V70del, V213G |

**Supplementary Table 9.** PRODIGY predicted binding energy of sialic acid to SARS-CoV-2 individual mutants, NIL – residue not covered by the structure. Red highlights are binding energy values for those variants which increase binding above 0.5 kcal/mol.

| Pocket | Wuhan-1 predicted binding energy | Mutations found in Variant of Concern/Interest | Mutant predicted binding energy |
| --- | --- | --- | --- |
| 1 | -6.6 kcal/mol | L18F (Beta, Gamma)  T19R (Delta, AY.1, AY.2, AY.3, B.1.617.3)  T19I (BA.2, BA.4, BA.5 Omicron)  D138Y (Gamma)  G142D (AY.1, AY2. B.1.617.3, Omicron)  Y144S, Y145N (B.1.621)  W152C (Epsilon)  E154K (Kappa)  E156G (AY.1) | -7.0 kcal/mol  -6.5 kcal/mol  -6.7 kcal/mol  -6.8 kcal/mol  -6.9 kcal/mol  -6.5 kcal/mol  -6.7 kcal/mol  -7.5 kcal/mol  -6.6 kcal/mol |
| 2 | -7.7 kcal/mol | L18F (Beta, Gamma)  T19R (Delta, B.1.617.3)  T20N (Beta, Gamma)  A67V (Eta)  G75V, T76I (Lambda)  D80A (Beta)  H245Y (B.1.620)  D253G (Iota)  D253N (Lambda)  W258L (AY.1, AY.2) | -7.8 kcal/mol  -7.4 kcal/mol  -7.8 kcal/mol  -7.4 kcal/mol  -7.4 kcal/mol  -7.6 kcal/mol  -7.6 kcal/mol  NIL  NIL  -7.4 kcal/mol |

**Supplementary Table 10.** PRODIGY predicted binding energy of sialic acid to SARS-CoV NTD pockets. Strain with an increased binding affinity of ≥0.5 kcal/mol as compared to the original Wuhan-1 strain of SARS-CoV-2 is coloured red.

| Virus | PRODIGY predicted binding energy (kcal/mol) | | |
| --- | --- | --- | --- |
|  | Pocket 1 | Pocket 2 | Pocket 3 |
| SARS-CoV | -5.5 | -6.4 | -6.6 |
| SARS-CoV-2 | -6.6 | -7.7 | -7.4 |
| SARS-CoV-2, B.1.117, Alpha variant | -6.4 | -7.4 | -7.0 |
| SARS-CoV-2, P.1, Gamma variant, EPI_ISL_804832 | -6.7 | -7.3 | -7.6 |
| SARS-CoV-2, P.1, Gamma variant, EPI_ISL_1219135 | -7.2 | -7.6 | -7.9 |
| SARS-CoV-2, P.1, Gamma variant, EPI_ISL_1068256 | -7.1 | -7.1 | -7.0 |
| SARS-CoV-2, B.1.351, Beta variant | -6.4 | -7.3 | -7.3 |
| SARS-CoV-2, B.1.617.1, Kappa variant | -6.8 | -7.4 | -7.6 |
| SARS-CoV-2, B.1.617.2, Delta variant | -7.1 | -7.5 | -7.0 |
| SARS-CoV-2, AY.1, Delta Plus variant | -6.6 | -7.6 | -6.7 |
| SARS-CoV-2, AY.2, Delta Plus variant | -7.1 | -7.7 | -6.9 |
| SARS-CoV-2, AY.3, Delta Plus variant | -6.6 | -7.5 | -7.7 |
| SARS-CoV-2, B.1.617.3 | -6.6 | -7.6 | -8.2 |
| SARS-CoV-2, B.1.429+B.1.427, Epsilon variant | -6.7 | -7.7 | -8.1 |
| SARS-CoV-2, B.1.525, Eta variant | -7.0 | -7.6 | -7.3 |
| SARS-CoV-2, B.1.526, Iota variant | -6.5 | -7.9 | -8.2 |
| SARS-CoV-2, B.1.620 | -6.9 | -7.9 | -7.4 |
| SARS-CoV-2, B.1.621, Mu variant | -6.9 | -6.9 | -7.8 |
| SARS-CoV-2, C.37, Lambda variant | -6.5 | -7.4 | -8.2 |
| SARS-CoV-2, P.3, Theta variant | -6.7 | -7.5 | -8.0 |
| SARS-CoV-2, N.10 | -6.4 | -6.8 | -8.0 |
| SARS-CoV-2, B.1.1.263 and BA.1, Omicron variant | -6.9 | -7.3 | -8.4 |
| SARS-CoV-2, BA.2, Omicron variant | -7.1 | -7.1 | -8.3 |
| SARS-CoV-2, BA.4, Omicron variant | -7.3 | -7.1 | -6.9 |
| SARS-CoV-2, BA.5, Omicron variant | -7.1 | -6.6 | -7.2 |

**Supplementary Table 11.** Impact of mutations in NTD and RBD is studied using the complex of SARS-CoV-2 spike protein with human ACE2 receptor (PDB ID: 7a95). Mutation S477N found in the Omicron variant with an increased binding affinity of ≥ 0.5 kcal/mol is coloured red.

| Variants of Concern  (VOC) | Domain | Mutation | mCSM-PPI2  prediction | Affinity  prediction |
| --- | --- | --- | --- | --- |
| Alpha (B.1.1.7) | NTD | S98F | 0.20 kcal/mol | Increasing |
| Alpha (B.1.1.7) | NTD | D138H | 0.20 kcal/mol | Increasing |
| Beta (B.1.351) | NTD | L18F | 0.20 kcal/mol | Increasing |
| Beta (B.1.351) | NTD | T19I | 0.08 kcal/mol | Increasing |
| Beta (B.1.351) | NTD | A27S | 0.15 kcal/mol | Increasing |
| Beta (B.1.351) | RBD | P384L | 0.10 kcal/mol | Increasing |
| Gamma (P.1) | NTD | L18F | 0.19 kcal/mol | Increasing |
| Gamma (P.1) | NTD | D138Y | 0.38 kcal/mol | Increasing |
| Gamma (P.1) | NTD | R190S | 0.10 kcal/mol | Increasing |
| Delta (B.1.617.2) | NTD | T19R | 0.08 kcal/mol | Increasing |
| Delta (B.1.617.2) | NTD | R158G | 0.09 kcal/mol | Increasing |
| Delta (B.1.617.2) | NTD | A222V | 0.28 kcal/mol | Increasing |
| Delta (B.1.617.2) | RBD | T478K | 0.03 kcal/mol | Increasing |
| Omicron (BA.1) | NTD | A67V | 0.27 kcal/mol | Increasing |
| Omicron (BA.1) | RBD | G339D | 0.32 kcal/mol | Increasing |
| Omicron (BA.1) | RBD | S371L | 0.10 kcal/mol | Increasing |
| Omicron (BA.1) | RBD | S375F | 0.14 kcal/mol | Increasing |
| Omicron (BA.1) | RBD | N440K | 0.28 kcal/mol | Increasing |
| Omicron (BA.1) | RBD | S477N | 0.57 kcal/mol | Increasing |

**Supplementary Table 12**. Summary of literature reporting sugar-binding pockets in N-terminal Domain of SARS-CoV-2

| **Pocket** | **Evidence** | **Reference** |
| --- | --- | --- |
| **Pocket 1** | 1. Docking and Molecular dynamics (MD) simulation using NTD and sialic acid 2. Multiple sequence alignment of the NTD- region:111–162 residues, using 11 isolates of SARS-CoV-2 | Fantini et al. 2020 [1] |
|  | 1. Docking, MD simulation, druggable pocket analysis and sequence alignment and structure modelling using SARS-CoV-2 as well as all VOCs/VOIs | This study |
| **Pocket 2** | 1. MD simulation using sialic acid | Awasthi et al. 2020 [8]; Bò et al. 2021[9] |
|  | 1. NMR technique (universal saturation transfer analysis) | Buchanan et al. 2022 [6] |
|  | 1. Glyconanoparticle platform | Baker et al. 2021 [5] |
|  | 1. Docking, MD simulation, druggable pocket analysis and sequence alignment and structure modelling using SARS-CoV-2 as well as all VOCs/VOIs | This study |
| **Pocket 3** | 1. MD simulation using NTD and sialic acid | Bò et al. 2021[9] |
|  | 1. Druggable pocket analysis using SiteMap | Di Gaetano et al. 2021[7] |
|  | 1. Docking, MD simulation, druggable pocket analysis and sequence alignment and structure modelling using SARS-CoV-2 as well as all VOCs/VOIs | This study |
| **Pocket 4** | Docking, druggable pocket analysis and sequence alignment and structure modelling using SARS-CoV-2 as well as all VOCs | This study |

Reference:

[1] Fantini J, Di Scala C, Chahinian H, Yahi N. Structural and molecular modelling studies reveal a new mechanism of action of chloroquine and hydroxychloroquine against SARS-CoV-2 infection. Int J Antimicrob Agents 2020;55:105960. https://doi.org/10.1016/j.ijantimicag.2020.105960.

[2] Cheng Y, He B, Yang J, Ye F, Lin S, Yang F, et al. Crystal structure of the S1 subunit N-terminal domain from DcCoV UAE-HKU23 spike protein. Virology 2019;535:74–82. https://doi.org/10.1016/j.virol.2019.06.015.

[3] Behloul N, Baha S, Shi R, Meng J. Role of the GTNGTKR motif in the N-terminal receptor-binding domain of the SARS-CoV-2 spike protein. Virus Res 2020;286:198058. https://doi.org/10.1016/j.virusres.2020.198058.

[4] Tortorici MA, Walls AC, Lang Y, Wang C, Li Z, Koerhuis D, et al. Structural basis for human coronavirus attachment to sialic acid receptors. Nat Struct Mol Biol 2019;26:481–9. https://doi.org/10.1038/s41594-019-0233-y.

[5] Baker AN, Richards S-J, Guy CS, Congdon TR, Hasan M, Zwetsloot AJ, et al. The SARS-COV-2 Spike Protein Binds Sialic Acids and Enables Rapid Detection in a Lateral Flow Point of Care Diagnostic Device. ACS Cent Sci 2020;6:2046–52. https://doi.org/10.1021/acscentsci.0c00855.

[6] Buchanan CJ, Gaunt B, Harrison PJ, Yang Y, Liu J, Khan A, et al. Pathogen-sugar interactions revealed by universal saturation transfer analysis. Science 2022:eabm3125. https://doi.org/10.1126/science.abm3125.

[7] Di Gaetano S, Capasso D, Delre P, Pirone L, Saviano M, Pedone E, et al. More Is Always Better Than One: The N-Terminal Domain of the Spike Protein as Another Emerging Target for Hampering the SARS-CoV-2 Attachment to Host Cells. Int J Mol Sci 2021;22:6462. https://doi.org/10.3390/ijms22126462.

[8] Awasthi M, Gulati S, Sarkar DP, Tiwari S, Kateriya S, Ranjan P, et al. The Sialoside-Binding Pocket of SARS-CoV-2 Spike Glycoprotein Structurally Resembles MERS-CoV. Viruses 2020;12:E909. https://doi.org/10.3390/v12090909.

[9] Bò L, Miotto M, Di Rienzo L, Milanetti E, Ruocco G. Exploring the Association Between Sialic Acid and SARS-CoV-2 Spike Protein Through a Molecular Dynamics-Based Approach. Front Med Technol 2021;2:614652. https://doi.org/10.3389/fmedt.2020.614652.
